# Bayesian classification of residues associated with protein functional divergence: Arf and Arf-like GTPases

**DOI:** 10.1186/1745-6150-5-66

**Published:** 2010-12-03

**Authors:** Andrew F Neuwald

**Affiliations:** 1 Institute for Genome Sciences and Department of Biochemistry & Molecular Biology, University of Maryland School of Medicine, BioPark II, Room 617, 801 West Baltimore St. Baltimore, MD 21201, USA

## Abstract

**Background:**

Certain residues within proteins are highly conserved across very distantly related organisms, yet their (presumably critical) structural or mechanistic roles are completely unknown. To obtain clues regarding such residues within Arf and Arf-like (Arf/Arl) GTPases--which function as on/off switches regulating vesicle trafficking, phospholipid metabolism and cytoskeletal remodeling--I apply a new sampling procedure for comparative sequence analysis, termed multiple category Bayesian Partitioning with Pattern Selection (mcBPPS).

**Results:**

The mcBPPS sampler classified sequences within the entire P-loop GTPase class into multiple categories by identifying those evolutionarily-divergent residues most likely to be responsible for functional specialization. Here I focus on categories of residues that most distinguish various Arf/Arl GTPases from other GTPases. This identified residues whose specific roles have been previously proposed (and in some cases corroborated experimentally and that thus serve as positive controls), as well as several categories of co-conserved residues whose possible roles are first hinted at here. For example, Arf/Arl/Sar GTPases are most distinguished from other GTPases by a conserved aspartate residue within the phosphate binding loop (P-loop) and by co-conserved residues nearby that, together, can form a network of salt-bridge and hydrogen bond interactions centered on the GTPase active site. Residues corresponding to an N-[VI] motif that is conserved within Arf/Arl GTPases may play a role in the interswitch toggle characteristic of the Arf family, whereas other, co-conserved residues may modulate the flexibility of the guanine binding loop. Arl8 GTPases conserve residues that strikingly diverge from those typically found in other Arf/Arl GTPases and that form structural interactions suggestive of a novel interswitch toggle mechanism.

**Conclusions:**

This analysis suggests specific mutagenesis experiments to explore mechanisms underlying GTP hydrolysis, nucleotide exchange and interswitch toggling within Arf/Arl GTPases. More generally, it illustrates how the mcBPPS sampler can complement traditional evolutionary analyses by providing an objective, quantitative and statistically rigorous way to explore protein functional-divergence in molecular detail. Because the sampler classifies the input sequences at the same time, it can be used to generate subgroup profiles, in which functionally-divergent categories of residues are annotated automatically.

**Reviewers:**

This article was reviewed by Frank Eisenhaber, L Aravind and Daniel Gaston (nominated by Eric Bapteste). For the full reviews, go to the Reviewers' comments section.

## Background

Phosphate-binding loop (P-loop) GTPases bind to guanine nucleotide via amino acid residues that show up at the sequence level as highly conserved motifs [1]. These motifs include: (i) a Walker A (Gx_4_GK[ST]) motif [2] corresponding to the P-loop [3], which binds to guanine nucleotide phosphate groups; (ii) a Walker B (DxxG) motif, the conserved glycine (G) and aspartate (D) of which (respectively) sense the γ phosphate of GTP and bind (via a water molecule) to the Mg++ ion that coordinates with guanine nucleotide phosphate groups; and (iii), within most of these proteins, a conserved threonine residue that likewise coordinates with the Mg++ ion.

**Figure 1:**
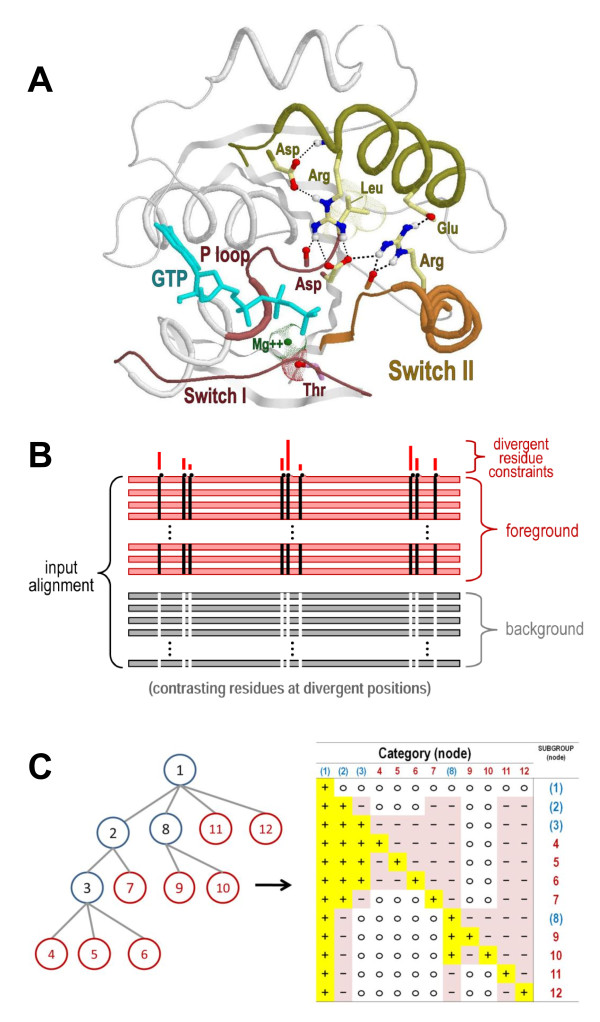
**Structure and analysis of Arf/Arl GTPases**. **(A) **Key structural features shared by Arf/Arl/Sar GTPases. In yellow are the side chains of six residues that are highly distinctive of Arf/Arl/Sar GTPases and that, within the GTP-bound state, often form the network of interactions shown. Within the Switch I region is shown a threonine residue (magenta side chain) that is conserved in the TRAFAC subclass of P-loop GTPases and that coordinates with the Mg++ ion associated with bound GTP. The structure shown is that of Arf1 bound to GTP (pdb_id: 1o3y; 1.50 Ǻ resolution) [57]. The Switch I and II regions (red and orange backbones, respectively) and the α3 helix (dark yellow backbone) are indicated. **(B) **Schematic representation of a contrast alignment, which reveals the sequence features distinguishing one group of proteins (termed the foreground) from related, evolutionarily-divergent proteins (termed the background). Red and gray horizontal bars represent sequences belonging to the foreground and background, respectively. Black vertical bars represent residues that are conserved in the foreground and white vertical bars represent corresponding non-conserved positions in the background. The histogram above these columns indicates the relative strengths of the selective constraints associated with evolutionary divergence of the foreground sequences from the background sequences. **(C) **Correspondence between a phylogenetic tree and a hyperpartition. A rooted tree is shown both as a graph and as a hyperpartition. In the hyperpartition, each column corresponds to one node in the tree such that the '+' rows in that column correspond to that node's subtree, which serves as the foreground, whereas the '-' rows in that column correspond to the rest of the parent node's subtree, which serves as the background; the remaining (non-participating) nodes in that column are labeled with an 'o'. Internal nodes (shown in blue) correspond to 'miscellaneous' subgroups, that is to sequences that are assigned to a subtree (e.g., a family), but not to a leaf node (e.g., a specific subfamily).

An important subgroup of P-loop GTPases is the Ras-like superfamily [4] (termed here the Ras-like GTPases), members of which function as signaling pathway on-off switches. A key characteristic of eukaryotic Ras-like GTPases is their association both with guanine nucleotide exchange factors (GEFs), which turn them on by mediating the exchange of GTP for GDP, and with GTPase activating proteins (GAPs), which turn them off by promoting the hydrolysis of GTP to GDP. GAPs and GEFs typically occur within multi-domain proteins that, together with Ras-like GTPases and other associated proteins, function as integrated circuits [5] to orchestrate cellular processes in response to environmental and developmental signals. When Ras-like GTPases are activated in response to appropriate upstream signals, they associate with various 'effectors' that propagate signals to downstream components of the pathway; when deactivated these signals are terminated. The GTPase on- or off-state is communicated to downstream components through conformational changes within the so-called switch I and II regions, which detect the presence or absence of the γ phosphate of bound guanine nucleotide (Figure 1A). Three major subgroups of Ras-like GTPases are (i) the Ras, Rab, Rac/Rho and Ran GTPases, (ii) the Arf, Arf-like (Arl), and Sar GTPases, and (iii) α subunits of heterotrimeric G proteins [6].

The existence of mysterious, as yet unidentified molecular mechanisms associated with Arf/Arl GTPases is suggested by the fact that they strikingly conserve specific residues whose functions, thus far, are completely unknown. Often such residues are shared by GTPases that have diverged from a common ancestor more than a billion years ago and that vary in their interactions with other cellular components, in their kinetics and localization, in their cellular functions and other properties--suggesting that the roles of these residues transcend functions or properties specific to a particular protein and instead reflect general mechanisms shared by otherwise dissimilar proteins. P-loop GTPases have diverged into multiple 'functional states', each of which is subject to somewhat different selective pressures due to their specialized cellular roles and mechanisms. Hence, the conservation of certain divergent residues within each subgroup reflects important structural or mechanistic distinctions between these subgroups.

Here a new Bayesian statistical approach is used to infer, from patterns of sequence conservation and divergence, various categories of co-conserved residues associated with Arf/Arl GTPase functional divergence. This approach is based upon an earlier procedure, termed Bayesian Partitioning with Pattern Selection (BPPS) [7,8]. The BPPS procedure partitions a multiple sequence alignment into two, functionally divergent subgroups by identifying a pattern that is conserved in one of the subgroups (termed the foreground), but that is non-conserved in the other subgroup (termed the background). In principle, it does this by first arbitrarily partitioning the input sequence alignment into foreground and background sub-alignments and by choosing an arbitrary pattern associated with the foreground sequences. (In practice, heuristics are used to speed up the procedure by finding a more meaningful starting point. Moreover, in order to focus on a particular protein of interest, either one sequence or a few closely-related sequences are pre-assigned to the foreground partition; such seed sequences serve a role analogous to that of the query in a database search.) Next an iterative strategy, called Gibbs sampling [9], is used to reassign sequences to either the foreground or background partition and to add or remove pattern residues with probability proportional to the degree to which such changes improve the underlying statistical model [7]. This process continues until convergence on an optimum or nearly optimum 'partition-pattern pair', which corresponds to two groups of proteins that have diverged from each other at pattern positions, as is shown schematically in Figure 1B.

The procedure used here generalizes this single category BPPS sampler into a multiple category (mc)BPPS sampler by introducing the notion of a *hyperpartition*, which consists of an *N *× *M *table of symbols and which is best understood by converting a phylogenetic tree into the corresponding hyperpartition, as is illustrated in Figure 1C. From this phylogenetic perspective: there are an equal number of rows and columns (i.e., *N *= *M*); each row (*i*) or column (*j*) corresponds to a specific node of the tree; and the table's symbols indicate the phylogenetic relationships between nodes. If node *i *belongs to the subtree starting from node *j*, then the cell in row *i *and column *j *contains a '+'; if it belongs to a sibling subtree, then the cell contains a '**-**'; and if it is somewhere else in the tree, then the cell contains an '**o**'. In other words, for each column, the '**+**' rows correspond to a clade that shares a most recent common ancestor with the clade or clades indicated by the '**-**' rows.

From the mcBPPS sampler's perspective, however, the *N *rows correspond to *N *distinct subgroups, and the *M *columns to *M *distinct BPPS analyses, where each analysis assigns each of the *N *protein subgroups either to one of two functionally-divergent groups--that is, to a foreground ('**+**') or background ('**-**') partition--or to neither group--that is to a third, 'non-participating sequence' ('**o**') partition. Based on these assignments, the sampler optimally assigns the input sequences to the *N *subgroups (the rows) and, at the same time, finds *M *patterns that optimally differentiate the foreground from the background (based on the hyperpartition specification) for each of the *M *functionally-divergent categories (the columns).

Although the phylogenetic relationships between subgroups typically are used to create a hyperpartition, strict conformity to a phylogenetic tree is not required. Subgroups of proteins from different clades can, in principle, share certain biochemical properties inherited from an ancestor of both clades, but lost in other members of both clades due to a relaxation of selective constraints upon them. Thus the hyperpartition allows arbitrary comparisons between groups, as long as certain restrictions are met--such as that each row (i.e., subgroup) includes at least one foreground assignment, that no two rows share identical foreground assignments, and that *M *≥ *N*.

Each subgroup is associated with a 'seed alignment' consisting of one or more sequences (typically no more than a dozen) that are known to belong to and that thus help define that subgroup (by serving as Bayesian priors). The seed sequences are required to remain in their assigned subgroups while the sampler iteratively reassigns the remaining sequences to subgroups and adds or removes pattern residues with probability proportional to the degree to which the evolving patterns distinguish the foreground from the background sequences for each category. At the same time, it automatically removes sequences that otherwise tend to obscure an analysis by explicitly modeling miscellaneous categories and aberrant sequences (i.e., pseudogene products and other non-functional homologs). The mcBPPS sampler thus sets up a stringent competition between the multiple categories of functional constraints imposed on the input sequences to thereby more precisely define both those co-conserved residues most distinctive of and those sequences belonging to each subgroup. Given our focus here, this identifies key residues associated with Arf/Arl GTPases functional divergence.

## Results and Discussion

**Figure 2:**
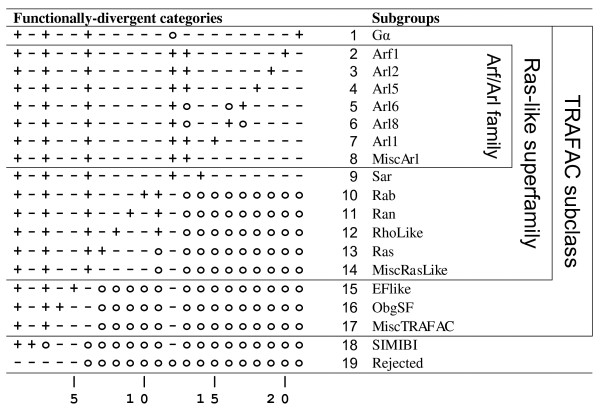
**Hyperpartition for P-loop GTPases with an emphasis on Arf/Arl GTPases**. The symbol '+', '-' or 'o' indicates that the subgroup is assigned to that category's foreground, background, or non-participating-sequence partition, respectively. Miscellaneous subgroups are italicized. Rejected sequences are those sampled into a random sequence set.

The mcBPPS analysis that is described here is based on the P-loop GTPase hyperpartition given in Figure 2. For each row (subgroup) of this hyperpartition, the mcBPPS procedure outputs 'contrast alignments'--one alignment for each column in that row that is assigned to the foreground ('**+**') partition. The full set of contrast alignments that were generated by the sampler in this way and that are most relevant to Arf/Arl GTPase functional divergence are given in Additional File 1. In the following sections we explore and interpret these alignments in the light of available structural and biochemical information.

### Features that Arf/Arl/Sar share with other P-loop and Ras-like GTPases

**Figure 3:**
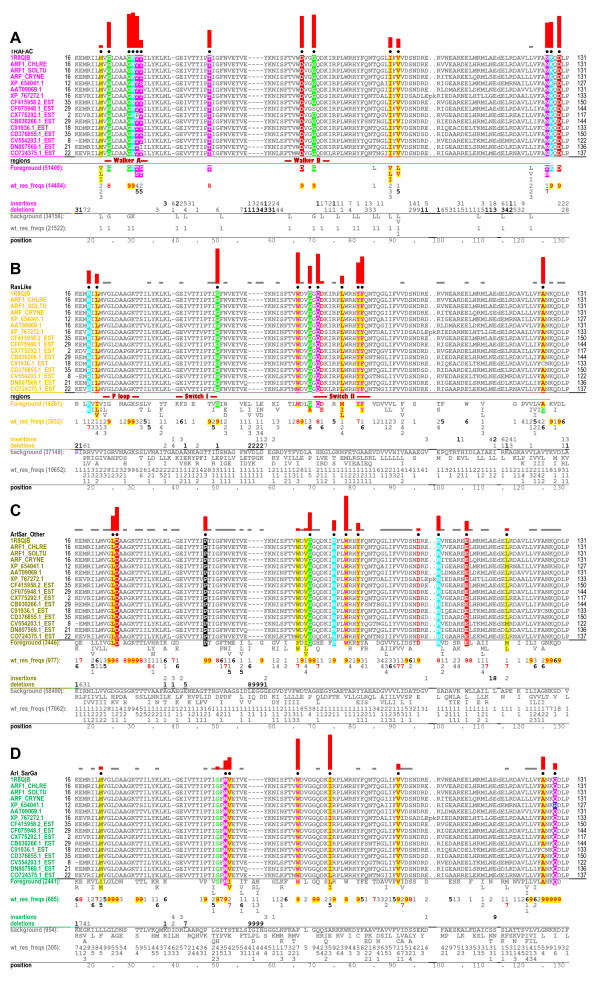
**Contrast alignments showing distinguishing sequence features of Arf/Arl GTPases**. This is the mcBPPS program output file corresponding to the Arf1-related subgroup (second row in Fig. 2); the sequences shown correspond to the seed alignment, which consists of Arf1 GTPases from distinct phyla. **(A) **Conserved residues distinguishing the TRAFAC subclass of P-loop GTPases from non-GTPases (column 3 in Fig. 2). **(B) **Conserved residues distinguishing Ras-like from other TRAFAC GTPases (column 6). **(C) **Conserved residues distinguishing Arf/Arl/Sar GTPases from other P-loop GTPases (except for Gα subunits)(column 12). **(D) **Conserved residues distinguishing typical Arf/Arl GTPases (i.e., excluding Arl6 and Arl8) from Sar and Gα GTPases (column 13). In each alignment, the Arf1 seed sequences are shown explicitly, whereas directly below these only the most conserved residue patterns in the foreground (relative to the background) are shown. Directly below this the corresponding weighted residue frequencies are shown (denoted by 'wt_res_freqs'). Weighted frequencies are given in integer tenths, where an '8', for example, indicates that the corresponding residue occurs in 80%-90% of the (foreground) sequences. Below this the conserved patterns and their weighted frequencies for the background sequences are shown (in gray). The selective constraints imposed on the foreground (relative to the background) at pattern positions are indicated by the histograms above each alignment.

The Arf1 contrast alignments shown in Figures 3A-B reveal (previously identified) co-conserved residue patterns characteristic of two major P-loop GTPase categories (within which are included Arf/Arl GTPases), namely transcription factor related (TRAFAC) [4] and Ras-like GTPases. The TRAFAC patterns [1] include the Walker A and B motifs (G-K-[ST] and D-x-x-G), a guanine binding loop motif ([NT]-K-x-D), and the switch I threonine residue (Thr48^Arf1^) that coordinates with a GTP-bound Mg++ ion. The Ras-like patterns include: (i) a switch II glutamine residue (Gln71^Arf1^) involved in GTP hydrolysis [10]; (ii) a glycine or alanine residue (Gly69^Arf1 ^in Figure 3B) within the Walker B motif (D-x-**G**-G); (iii) a glycine residue (Gly50^Arf1^) bordering the switch I region that may function as a hinge for switch I conformational change; (iv) five residues (Arg19^Arf1^, Leu21^Arf1^, Trp66^Arf1^, Tyr81^Arf1 ^and Phe82^Arf1^) that, though dispersed in the sequence, mutually interact near the switch II C-terminal region and that, within Rab and Ran GTPases, can form a "charge-dipole pocket" configuration that is associated with formation of an unusual, outward-oriented switch II α-helix [11]; and (v) a glycine or alanine residue (Ala125^Arf1^) that directly precedes the guanine binding loop ([**GA**]-[NT]-K-x-D). These patterns provide a sequence and structural context within which to better evaluate the following Arf/Arl functionally-divergent features.

### Features distinguishing Arf/Arl/Sar from other P-loop GTPases

**Figure 4:**
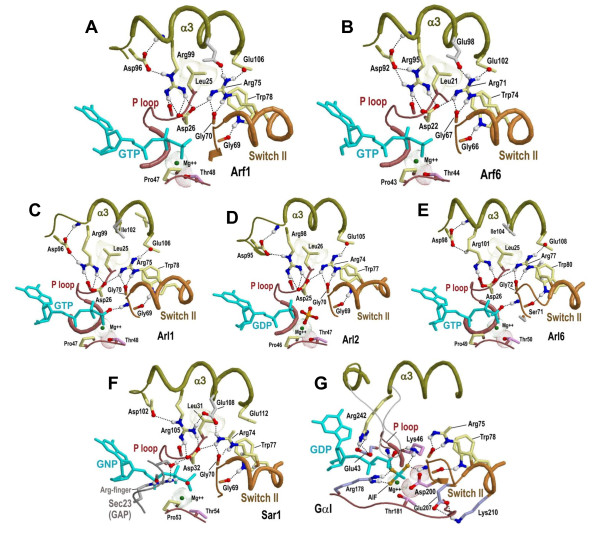
**Structural features associated with co-conserved residues that are distinctive of Arf/Arl/Sar GTPases and that form a network of salt bridges**. Arf/Arl/Sar GTPases features correspond to column 12 in Fig. 2 and to the contrast alignment in Fig. 3C. See the legend to Fig. 1A for side chain and backbone color schemes. Shown in gray are the side chains of residues at an (unclassified) position within the α3-helix that weakly conserves an acidic amino acid which also participates in the Arf/Arl salt-bridge network. (**A**) Arf1 + GTP (pdb_id: 1o3y; 1.50 Ǻ)[57]. (**B**) Arf6 + GTP + Cholera toxin (not shown) (pdb_id: 2a5d; 1.80 Ǻ) [36]. (**C**) Arl1 + GTP + Golgin-245 Grip domain (not shown) (pdb_id: 1upt; 1.7 Ǻ) [37]. (**D**) Arl2 + GDP + PO_4_^3- ^and its effector PDE-δ (not shown) (pdb: 1ksh; 1.80 Ǻ) [38]. (**E**) Arl6 + GTP (pdb_id: 2 h57; 2.00 Ǻ) [Structural Genomics Consortium]. (**F**) Sar1 GTPase bound to a GTP analog (GppNHp)) and to the GAP protein Sec23 (pdb_id: 1m2o; 2.50 Ǻ) [41]; the side chain of the arginine-finger of Sec23 is shown in gray. (**G**) Gα bound to RGS4, GDP and AlF_4 _(transition state structure)(pdb_id: 1agr; 2.8 Ǻ) [58]. The side-chains of three residues that are specifically conserved in Gα subunits[6] (one of which is the arginine finger, Arg178) are shown in blue.

Figure 3C highlights the conserved residues that most distinguish Arf/Arl and Sar GTPases from other P-loop GTPases. For this category, heterotrimeric G protein α subunits were omitted because they share some, but not all Arf/Arl/Sar characteristic features [6] (see below), which thus tends to obscure the analysis. These features correspond to about a dozen residue positions, five of which typically form--within the GTP-bound state--a network of salt bridge interactions (Figure 4A-F) centered on an aspartate residue within the P-loop (Asp26^Arf1 ^in Figures 3C and 4A) and on the Walker B glycine residue (Gly70^Arf1^).

#### An Arf/Arl/Sar salt bridge network

The previously noted [12-14] conserved P-loop aspartate (Asp26 in Figure 4A) appears to be a key residue within the Arf/Arl salt bridge network. Within Ras GTPase, this residue position corresponds to a P-loop glycine (Gly12 in H-Ras), mutation of which to any other amino acid except proline inhibits GTP hydrolysis and thus leads to a constitutively active GTP-bound state [15] that promotes oncogenesis by continually up-regulating downstream pathways. Moreover, a glycine to aspartate mutation is one of the most frequent oncogenic mutations associated with the K-ras gene in humans [16,17] and, indeed, is the most frequent mutation found in human tumors [18]. Rho, Ran and Gα family GTPases also typically conserve a glycine at this position and, among all P-loop GTPases, aspartate very rarely occurs at this position outside of the Arf, Arl and Sar families--a notable exception being the dynein light intermediate chain family. Less than 0.1% (224 out of 258,895) of the remaining P-loop GTPases identified in this analysis harbor an aspartate at this position.

Within many crystal structures of Arf/Arl/Sar GTPases (Figure 4A-F), the P-loop aspartate forms hydrogen bonds with two co-conserved arginine residues--one in the Switch II region and another associated with the α3 helix (Arg75^Arf1 ^and Arg99^Arf1^, respectively, in Figure 4A). Each of these arginine residues, in turn, forms hydrogen bonds with two other co-conserved acidic residues, both of which occur within or near the α3 helix (Asp96^Arf1 ^and Glu106^Arf1^, respectively). The switch II arginine residue (e.g., Arg75^Arf1^) also typically hydrogen bonds to the backbone oxygen of the Walker B (D-x-x-G) motif glycine residue (e.g., Gly70^Arf1^); this glycine senses the γ-phosphate group of bound GTP via its backbone NH group. A co-conserved tryptophan residue within the Switch II region (e.g., Trp78^Arf1^) likewise forms a hydrogen bond to the backbone oxygen of another co-conserved glycine residue (Gly69^Arf1^); the backbone C = O group of this glycine participates in a peptide bond with the γ-phosphate-sensing backbone NH group of the Walker B glycine. The tryptophan also packs up against the Arf/Arl/Sar Switch II arginine and, in many GTP-bound structures, appears to form two CH-π interactions with it. These three co-conserved residues (i.e., the tryptophan, glycine and arginine) and their structural interactions were noted previously [19]. Finally, the side chain of a co-conserved leucine residue (Leu25-Arf1 in Figures 3C and 4A) that directly precedes the P-loop aspartate packs up against the two arginine residues and against the glutamate and tryptophan residues--suggesting that it may facilitate the associated hydrogen bond and salt bridge interactions by helping to position these residues sterically. Thus a common theme shared by all of these residues is that they converge on regions that are in close proximity to the γ-phosphate of bound GTP.

Notably, a mcBPPS analysis of the retinitis pigmentosa 2 (RP2) gene product (Additional File 2), which functions as a GTPase-activating protein for Arl3 GTPase [20], suggests that interactions between RP2 co-conserved residues and residues of the Arf/Arl/Sar-salt bridge network within Arl3 may help stabilize the transition state for GTP hydrolysis.

#### Similarities with Gα subunits

The switch II tryptophan-arginine residue pair characteristic of Arf/Arl/Sar GTPases is also conserved in heterotrimeric G protein α subunits. In Gα these residues also form similar structural interactions within the GTP-bound state [6] (Figure 4G). Likewise, Gα subunits conserve the α3 helix arginine residue, though not the P-loop aspartate residue that, within Arf/Arl/Sar GTPases, interacts with this arginine. Instead, Gα subunits conserve the P-loop glycine that is conserved in Ras, as well as a P-loop glutamate residue that directly follows (in sequence) this P-loop glycine and that can form a salt bridge interaction with the α3 arginine. Thus Gα and Arf/Arl/Sar GTPases both conserve the potential for forming a salt bridge between a P-loop acidic residue and an α3 helix arginine residue.

#### The α3 helix salt bridge network versus a 'glycine brace'

Within Ran, Rab and Rho GTPases, the α3 helix and the loop preceding it lack the characteristic Arf/Arl/Sar co-conserved residues and instead are associated with a different set of co-conserved residues: two aromatic residues (Phe91^Rab11 ^and Trp105^Rab11 ^in Fig. S1C of Additional File 1) that form CH-π and NH-π interactions with two putative glycine hinge points for the P-loop and for the guanine binding loop, and an aspartate residue (Asp19^Rab11^) within the P-loop region that, along with a co-conserved serine/threonine residue (Thr98^Rab11^) and a buried conserved water molecule, form a network of interactions between the two aromatic residues. This configuration was termed a glycine brace whose proposed role is to stabilize the guanine nucleotide binding loops [21]. Thus the analysis here indicates that Arf/Arl/Sar and Rab/Ran/Rho GTPases exhibit distinct structural motifs--a salt bridge network and a glycine brace, respectively--that establish interactions linking the region preceding and including the α3 helix to regions that directly interact with bound guanine nucleotide. It is worthwhile noting in this context that a weak distinguishing feature of Arf/Arl/Sar/Gα GTPases (which requires a low contrast setting to show up) is an aromatic residue (Phe90 in Arf1) that, within some structures, appears to form a CH-π interaction with the glycine residue (Gly24^Arf1^) corresponding to one of the glycines stabilized by the brace.

#### A possible membrane-associated tryptophan residue

An Arf/Arl/Sar co-conserved tryptophan within the C-terminal end of the GTPase domain (Trp172-Arf1 in Fig. S2C of Additional File 1) is located structurally near the N-terminal myristate moiety [22]--a posttranslational modification implicated in membrane association and a feature required for the biological function of Arf GTPases. Given tryptophan's inherent affinity for the lipid bilayer [23-25], this residue might play a role in membrane association.

### Distinguishing sequence features of Arf/Arl versus Gα and Sar GTPases

**Figure 5:**
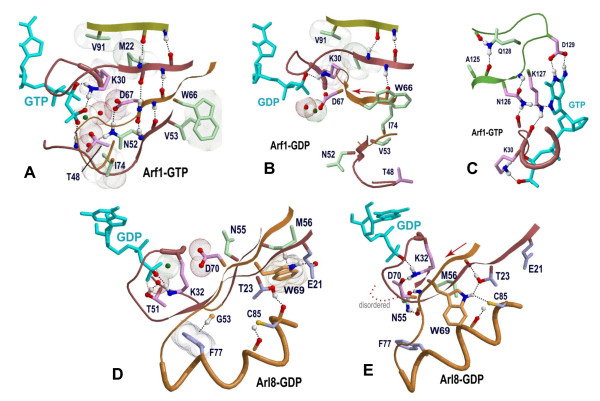
**Structural locations of Arf/Arl co-conserved residues**. (**A-C**) Conserved residues (green side-chains) distinguishing Arf/Arl from Sar and Gα GTPases (column 13 in Fig. 2; Fig. 3D). **(A) **The structure of Arf1 bound to GTP. The region shown corresponds to a β sheet adjacent to the Walker B region. Note that the asparagine of the N-[VI] motif (Asn52) forms a hydrogen bond with the Walker B aspartate (Asp67), whereas the valine (Val53) packs up against a tryptophan (Trp66) that directly precedes the aspartate. An Arf/Arl-conserved isoleucine (Ile74) packs against the backbone connecting the switch I threonine residue (Thr48) to the switch I glycine (Gly50; unlabeled in figure). **(B) **The structure of Arf1 bound to GDP. Note that the β-strand corresponding to the Walker B "W-D" motif has shifted to the left by two residue positions due to Arf's interswitch toggle and that the N-[VI] motif interactions are disrupted. **(C) **The structural location of the Arf/Arl conserved glutamine (Gln128) and alanine (Ala125) residues associated with the guanine binding loop (the NK-x-D motif). The glutamine may stabilize this loop by forming hydrogen bonds with backbone oxygen atoms on either side of this loop, as shown. **(D-E)**. Structural locations of residues (blue side-chains) that are conserved in and distinctive of Arl8 GTPases (column 16 in Fig. 2; Fig. S4D in Additional File 1). Residue side chains corresponding to the N-[IV] motif (of Arf/Arl GTPases) are shown in green. Note that Arl8-specific conserved residues form alternative interactions in the two forms, suggesting that they play a role in the interswitch toggle. **(D) **A non-interswitch toggled form of Arl8-GDP (pdb_id: 1zd9; structural genomics consortium). **(E) **An interswitch toggled form of Arl8-GDP (pdb_id: 2 h18; structural genomics consortium).

The mcBPPS analysis also identifies sequence features that most distinguish Arf/Arl from Gα and Sar (column 13 in Figure 2; Figure 3D) (this comparison excludes Arl6 and Arl8 GTPases because they lack many features typical of Arf/Arl GTPases; see below). Among the most strikingly divergent residues is a tryptophan (Trp66^Arf1^) that is also categorized as a Ras-like co-conserved residue (Figure 3B); it shows up in this category as well because within Arf/Arl this residue is nearly always a tryptophan, whereas Gα and Sar nearly always conserve the smaller aromatic residues, phenyalanine or tyrosine, instead. Within available structures of GTP-bound Arf/Arl GTPases, this tryptophan packs up against and may form a CH-π interaction [26] with the valine or isoleucine residue of a N-[IV] motif (Val53^Arf1 ^in Figures 3D and 5A), while the asparagine of this motif (Asn52^Arf1^) forms a side-chain hydrogen bond with the Walker B aspartate (Asp67^Arf1 ^in Figures 3D and 5A), which is sequence-adjacent to the tryptophan (i.e., the Walker B region conserves the pattern W-D-x-x-G).

However, for Arf/Arl GTPases in the GDP-bound state, the β-strand containing this tryptophan and the adjacent Walker B aspartate is typically displaced by two residue positions relative to the adjacent β-strand (Figure 5B), which lead Pasqualato *et al. *[19] to propose an 'interswitch toggle' device for front-to-back communication from the N-terminus to the nucleotide binding site in Arf/Arl/Arp/Sar GTPases. Moreover, Pasqualato *et al. *proposed that movement of the aspartate of the DxxG motif mimics the charge of the γ-phosphate of GTP to thereby prevent binding of GTP. Thus, the side chain interactions between the residues of the W-D and N-[IV] motifs within the GTP-bound state may help counteract the tendency for this region to toggle forward. Indeed, it is the backbone β-strand interaction between residues of the W-D motif and the N-[IV] motif that is shifted by two positions when the interswitch region toggles, and, in the GDP-bound toggled state, the interactions between the N-[IV] motif residues and the tryptophan and aspartate residues are completely disrupted. Arl6 GTPases are atypical inasmuch as they conserve neither the tryptophan nor the N-[IV] motif, and, unlike typical Arf/Arl/Sar GTPases, they conserve a serine instead of a glycine with the Walker B motif (D-x-**S**-G; Ser71^Arl6 ^in Figure 4E).

Another feature distinguishing Arf/Arl from Gα/Sar GTPases is an isoleucine or leucine residue (Ile74^Arf1 ^in Figure 5A) the side-chain of which, in the GTP-bound state, packs against the switch I backbone residue that is located between a conserved glycine residue and a conserved threonine residue. The glycine residue (Gly50^Arf1^) is conserved within Ras-like GTPases and may function as a hinge point, whereas the threonine residue (Thr48^Arf1^) coordinates with the Mg++ ion associated with guanine nucleotide phosphate groups. This conserved isoleucine or leucine residue is sequence adjacent to the switch II arginine (Arg75^Arf1^) that is conserved in Arf/Arl/Sar (and Gα) GTPases and that, in the GTP-bound state, forms a hydrogen bond with the backbone oxygen of the Walker B glycine residue which senses γ-phosphate. Thus, one possible explanation for co-conservation of this isoleucine/leucine residue with the N-[VI] motif is that they cooperatively stabilize the non-toggled GTP-bound state.

Sar GTPases also exhibit an interswitch toggle but fail to conserve any of these features (as was expected inasmuch as Sar GTPases were assigned to the background in column 13 of Figure 2). Moreover, unlike Arf/Arl GTPases, Sar GTPases lack two Ras-like canonical residues that are located near the γ-phosphate of bound GTP, namely (i) the Switch I glycine residue (Gly50^Arf1 ^in Figure 3B) that may function as a hinge point; and (ii) the catalytic glutamine residue (Gln71^Arf1 ^in Figure 3B) near the N-terminal end of the Switch II region. Indeed, what most distinguishes Sar GTPases from Arf/Arl and Gα GTPases (column 14 in Figure 2; Fig. S3D in Additional File 1) is the conservation of histidine instead of the Arf/Arl canonical residues at both of these positions and of a proline residue directly following the switch I histidine. This proline directly precedes the residue position that, within Arf/Arl GTPases, harbors the asparagine of the N-[VI] motif and that, within the non-toggled GTP-bound conformation, forms two backbone hydrogen bonds with the Walker B-aspartate. Taken together, this suggests that certain aspects of Sar's catalytic and toggling mechanisms fundamentally differ from that of typical Arf/Arl GTPases.

#### Alternative contexts for the guanine binding motif

Ras-like GTPases, with the exception of Gα subunits, conserve a glycine or alanine residue (Ala125^Arf1 ^in Figure 3B) directly before the guanine binding motif (i.e., [GA]-N-K-x-D). Ran, Rab, Rho and most Ras family GTPases conserve a glycine residue that appears to be stabilized by one of the two aromatic residues of the proposed glycine brace [21]. Sar GTPases also conserve this glycine, though they lack the conserved aromatic CH-π interaction. Arf/Arl GTPases conserve a (less flexible) alanine residue at this position. Notably nearly all other P-loop GTPases harbor larger residues at this position, suggesting that a small residue may be functionally necessary for Ras-like GTPases (other than Gα). Furthermore, at the x-position of the N-K-x-D motif, Arf/Arl GTPases (other than the atypical Arl6 and Arl8 GTPases) co-conserve a glutamine (e.g., Gln128^Arf1 ^in Figure 5C), the side chain of which can form hydrogen bonds with the backbone oxygen of the co-conserved alanine preceding this motif or of a residue near the end of the guanine binding loop or of both. Thus, these Arf/Arl residues (as well as the corresponding glycine brace residues) may help modulate the flexibility of the guanine binding loop and consequently its nucleotide affinity in response to upstream regulatory signals.

### Arl8 is an atypical Arf/Arl GTPase

Among Arf/Arl GTPases, the Arl8 subfamily (also termed Arl10B,C [27]) stands out as lacking several of the canonical residues characteristic of both Ras-like and other Arf/Arl GTPases (Figs. S4 in Additional File 1). **(i) **Arl8 GTPases lack the canonical [RK]-x-[LIV] N-terminal motif characteristic of Ras-like GTPases and, instead, conserve an [ED]-x-[TS] motif--a pattern that other atypical Ras-like GTPases lack. **(ii) **They currently are the only Ras-like GTPases for which a conserved cysteine substitutes for the second aromatic residue of the Y-[YF] motif within the switch II region. **(iii) **Instead of harboring an isoleucine/leucine residue prior to the Arf/Arl/Sar switch II R-x-W motif, Arl8 conserves (as do many non-Arl/Arf/Gα/Sar GTPases) a phenylalanine (Phe77^Arl8 ^in Figs S4D and 5D); this residue appears to form a CH-π interaction with the conserved glycine residue (Gly53^Arl8^) bordering the switch I region of Ras-like GTPases--thereby perhaps stabilizing this potential glycine hinge point. **(iv) **Arl8 GTPases conserve a glutamine instead of the P-loop aspartate typical of Arl/Arf/Sar GTPases and fail to conserve the arginine residue with which the P-loop aspartate interacts (not shown). And **(v) **they lack the conserved glutamine and alanine residues that are associated with the NKxD motif within typical Arf/Arl GTPases. For these reasons, Arl8 GTPases are very likely to differ mechanistically from other Arf/Arl GTPases and thus are perhaps best placed into a distinct category. Nevertheless, Arl8 GTPases do conserve certain Arf/Arl features such as the asparagine of the N-[VI] motif and a tryptophan directly preceding the Walker B aspartate.

Further insight into Arl8 divergence is obtained by examining available crystal structures. Even though a structure of Arl8 GTPases bound to GTP is not yet available, both interswitch toggle conformations are observed within available structures of Arl8 bound to GDP (Figures 5D, E). One of these corresponds to the form normally associated with the GTP-bound state (Figure 5D) inasmuch as the β-strand associated with the D-x-x-G motif forms the same hydrogen bonds with the adjacent β-strands (these bonds are not shown in Figures 5D, E). In this form, the Arl8 residue corresponding to the second position of the Arf/Arl N-[VI] motif, namely Met56, packs up against the tryptophan residue directly preceding the D-x-x-G motif, as does the corresponding isoleucine or valine within typical Arf/Arl GTPases (when in the GTP-bound state). However, unlike typical Arf/Arl GTPases, the indole amine of the tryptophan forms a hydrogen bond with the side chain oxygen of the Arl8-conserved glutamate residue (Glu21 in Figure 5D) that replaces the basic residue normally found at this position in Arf/Arl (and in other Ras-like) GTPases. At the same time, the Arl8-conserved phenylalanine residue (Phe77^Arl8 ^in Figure 5D) appears to form a CH-π hydrogen bond with the putative switch I glycine-hinge (Gly53^Arl8 ^in Figure 5D).

However, in a second, interswitch-toggled conformation (Figure 5E) the β-strand in which this tryptophan occurs is shifted toward the guanine nucleotide binding site by two positions and forms a hydrogen bond with a threonine residue (Thr23) that again is specifically conserved within Arl8 GTPases. The indole amine of this tryptophan may also form a NH...S hydrogen bond with a cysteine residue (Cys85) that likewise is specifically conserved within Arl8 GTPases. Although the atomic coordinates are slightly outside of the ideal range for N-H...S hydrogen bonds [28] (and the electron-density map is unavailable), previous studies support a propensity for the indole amine of tryptophan to form N-H...S hydrogen bonds [29,30]. Thus the threonine and cysteine residues may be conserved due to an Arl8-specific interswitch toggle mechanism.

## Conclusions

Here a mcBPPS analysis of Arf/Arl/Sar GTPases has provided clues regarding various co-conserved residues whose functions are currently unknown. Although by no means determining their functional roles, this provides guidance by generating plausible hypotheses for experimental follow up. For example, The N-[VI] motif asparagine within Arf/Arl GTPases could be mutated to an aspartate (a relatively minor replacement of the side-chain -NH_2 _group with an oxygen atom) to explore the influence of the -NH2 group on the interswitch toggle device and, as a result, perhaps on GEF or GAP activity. Mutation of non-canonical residues back to the canonical residues for atypical Arf/Arl GTPases could also be informative (and seem unlikely to introduce dramatic structural perturbations given their prevalence within typical Arf/Arl GTPases). For instance, mutation of the Switch II cysteine within Arl8 to the canonical tyrosine or phenylalanine may impair the ability of this GTPase to toggle if a potential NH...S interactions with the Walker B tryptophan is functionally important. Alternatively, replacing this cysteine with a serine (which never occurs at this position within available Arl8 family sequences) merely replaces the larger sulfur atom with a smaller oxygen atom.

Likewise, consider the Gα GTPase conserved arginine residue that is structurally homologous with the α3 helix arginine residue that forms a salt bridge with the P-loop aspartate within the Arf/Arl/Sar families. This Gα arginine likewise forms a salt bridge with a P-loop glutamate residue suggesting potential mechanistic similarities (and differences) with the Arf/Arl/Sar GTPases. Mutagenesis of the P-loop glutamate within Gα either to glutamine (thereby replacing its carboxylate side-chain group with a carboxamide group) or to aspartate (thereby shortening its sidechain by one carbon atom), seems likely to have subtle (and hence interpretable) effects that may provide insight into the role of this salt bridge interaction within Gα GTPases. A possible membrane-association role for the highly-surfaced-exposed tryptophan residue near the N-terminal myristate moiety (Trp172^Arf1 ^in Figure S2C) could also be explored through mutagenesis. Of course, these are but a few examples of the Arf/Arl/Sar divergent residues identified by the mcBPPS sampler that could be explored in this way.

More generally, this study illustrates how the mcBPPS sampler complements traditional evolutionary analyses by characterizing protein functional-divergence objectively and quantitatively, as opposed to "eye-balling" divergent residues within a few, manually-selected representative sequences. Several advantages of the sampler in this regard are worthwhile pointing out: It can reveal detailed molecular distinctions between divergent subgroups from multiple perspectives. It can be applied to a vast number of sequences. It precisely classifies the sequences into subgroups based on the multiple categories of differentiating patterns that it identifies. And it can automatically eliminate pseudogene products, unrelated sequences, and other non-functional or functionally impaired proteins by sampling them either into a 'rejected sequence subgroup' or into miscellaneous subgroups. Moreover, the sampler can compute predictive probabilities for sequence membership in various subgroups (and, by implication, for protein function). Finally it can be used to generate subgroup profiles, in which the various functionally-divergent categories of residues are annotated automatically. Version 1.0 of the mcBPPS program is freely available at http://www.chain.umaryland.edu/mcbpps.

## Methods

### Multiple category Bayesian Partitioning with Pattern Selection (mcBPPS)

Here a conceptual description of the mcBPPS sampler is provided. Mathematical and algorithmic details regarding the sampler, the distinctions between it and other methods, and an evaluation of its performance will be published elsewhere (Neuwald, submitted). The mcBPPS program requires three input files: (i) A (typically) large number of multiply aligned sequences belonging to the protein class of interest. (ii) An *N *× *M *table (termed a hyperpartition) that specifies which subgroups are assigned to the foreground and background for each of *M *'functional constraint categories'. And (iii) a 'seed alignment' for each of the *N*-1 subgroups modeled by the hyperpartition (a seed alignment is not required for an *N*-th subgroup, which consists of random sequences). Given this input, the mcBPPS sampler runs (in parallel) multiple BPPS procedures (one for each constraint category) both to optimally assign the input sequences to the subgroups (and, by implication, to the foreground and background partitions for each category) and to define, for each category, a pattern that optimally differentiates the foreground from the background sequences. The sampler outputs a set of 'contrast alignments' that reveal the co-conserved residue patterns characteristic of each subgroup; it also outputs those input sequences that have been assigned to each subgroup.

#### Input multiple sequence alignments

Multiple alignment of available GTPase sequences was accomplished using a previously described strategy [11] that iterates between sequence detection and alignment using the MAPGAPS program [31]http://mapgaps.igs.umaryland.edu. This yielded an alignment of 258,895 sequences. Sequence fragments and nearly-identical sequences (those sharing more than 98% sequence identity) were removed, yielding a final alignment consisting of 66,386 sequences. Seed alignments, which serve as Bayesian priors to help define each sequence set, were obtained by selecting from the main sequence alignment a few well-characterized proteins belonging to each of the major P-loop GTPase subgroups and to each of the largest Arf/Arl subfamilies. For certain subgroups, consensus sequences were used as seeds.

#### The hyperpartition

The relationships between distinct sequence subgroups within a protein class are specified using a hyperpartition, which consists of a *N *× *M *matrix that assigns *N *sequence subgroups to the foreground, background or non-participating-sequence partitions for each of the *M *functionally-divergent categories to be modeled. As described in the *Introduction*, each foreground partition consists of sequences that share certain co-conserved patterns and that have diverged from the background sequences lacking those patterns; the non-participating-sequences are excluded from the comparison. The hyperpartition defined here is given in Figure 2. The choice of which subgroups are assigned to which partitions for each category is, in some sense, arbitrary depending on what questions are being asked--though the mcBPPS procedure will reject categories that lack empirical support (i.e., that lack differentiating sequence patterns). Most often categories are defined to reflect the phylogenetic relationships between protein sequences (e.g., Arf subfamilies will be classified together as having diverged from a single ancestral Ras-like GTPase; Ras-like families will be classified as having diverged from a single ancestral P-loop GTPase; *et cetera*)(See Figure 1C).

### Structural analysis

Structural analysis of sequence features was performed using routines that have been implemented within the CHAIN program [32] and that were used to generate RasMol [33] scripts for each category of co-conserved residues identified by the mcBPPS program.

### Sequences and structures used in the analysis

Sequences were identified within the NCBI nr, env_nr and translated EST databases; only translated EST open reading frames of at least 100 residues in length were used. The pdb identifiers corresponding to the proteins structures shown in Figures 4 and 5 are: Arf1_human, 1R8SA [34]; Arf2_yeast, 1MR3F [35]; Arf5_human, 2B6HA (Structural Genomics Consortium (SGC)); Arf6_human, 2A5DA [36]; Arl1_human, 1UPTA [37]; Arl1_yeast, 1MOZA [35]; Arl2_mouse, 1KSHA [38]; Arl3_mouse, 1FZQA [39]; Arl8_human, 1YZGA (SGC); Arl5_human, 2H17A (SGC); Arl6_human, 2H57A (SGC); Arl10b_human, 1ZD9A (SGC); Sar1a_human, 2FMXA [40]; and Sar1_yeast, 1M2OB [41]. The Reduce program [42] was used to predict hydrogen atom positions within structural coordinate files; this step is required for automated detection of hydrogen bonds using the CHAIN program [32]. Molecular images were created using Rasmol [33].

## Competing interests

The author declares that they have no competing interests.

## Reviewer's comments

### Reviewer 1: Frank Eisenhaber (together with Sebastian Maurer-Stroh), Bioinformatics Institute, Agency for Science, Technology and Research, Singapore

Reviewer 1

Using an extension of his Bayesian classification algorithm, the author identifies and describes residues that differ among various GTPase families with particular focus on Arf and Arl-like GTPases. The analysis and method strongly depend on prior subfamily knowledge and alignment of pre-classified seed families which would make the same approach less easily applicable to other families, at least in an unsupervised manner.

***Author's response**: When used in conjunction with the original, single-category BPPS sampler, the mcBPPS sampler can readily be applied to unclassified protein superfamilies. Seed sequences serve a role analogous to that of the query in a database search: they allow the user to ask specific questions regarding proteins of interest*.

Reviewer 1

The seed dependency is not a major problem for this manuscript since the selection of pre-classified GTPase subfamilies is in good agreement with the literature in this field but simplifies the evolutionary scenario for some of the subfamilies, e.g. Galpha proteins, which could be further split into smaller families as was done for the different Arf/Arls. Maybe the difficulties reported when comparing Gα to Arf/Arls could be overcome with more attention given to detailed pairwise subfamily similarities/differences. In general, it would be interesting if the seed grouping and group relations could be automatically derived and weighted, for example from phylogenetic trees. If the author prefers probabilistic sampling, BEAST, would be an option to fit into the Bayesian framework.

***Author's response**: I have performed additional comparisons along the lines suggested, but--because this added little to the overall analysis and to keep the manuscript readable--these were left out. The mcBPPS program includes a routine to convert a phylogenetic tree into a hyperpartition. Thus, a phylogenetic tree could be used as suggested--though I have not fully automated this process*.

Reviewer 1

The input alignment used cuts out both N- and C-termini of the proteins which may have appeared messy in the large alignment due to strongly differing terminal extension lengths. However, besides having different lengths of linker regions, these termini are actually also characteristic for subfamilies. For example, several of the Arf/Arl subfamilies are either N-terminally myristoylated or acetylated which dramatically alters their cellular context and function. Similarly, some but not all Galpha subfamilies are also myristoylated, while Ras, Ran, Rho, Rab etc. have different subfamily-specific patterns of farnesylation or geranylgeranylation. A possible solution to the problem of aligning termini to be included in this analysis would be to assume that the respective motifs for posttranslational modifications are recognized as extended peptides followed by some linkers of varying length. The terminal portions of the alignment could, therefore, simply be assumed to be gapless while the linkers of varying lengths between the termini and the globular structure (current alignment) can be more or less ignored.

***Author's response**: There are, of course, various ways in which this sort of analysis could be performed. My inclination would be to perform a separate analysis of such N- and C-terminal extensions. One could do the same both for the large insert regions that occur in certain GTPases and for interacting protein domains*.

Reviewer 1

The Bayesian classification method used is not well described but builds mainly upon previous publications. For easier understandability, a few sentences could be added to give a bit more detail, especially on how conservation was initially derived/defined. For example, the manuscript mentions the importance of detecting positions fully conserved in the foreground but not conserved in the background set. However, subfamily-specific positions that are different between families may still be conserved within their respective subfamilies which makes them even more interesting for functional specificity but they will be different in terms of probabilities for drawing the same distribution by chance compared to random background sequences (e.g. one position with "all Lysine in Arf1 but all Glutamate in Arf2" would be different from "all Lysine in Arf1 and no conservation at all in Arf2").

**Author's response**: I have written a detailed description of the statistical formulation and algorithmic procedure within a separate, methods- and evaluation-oriented paper. This methods-oriented paper explicitly defines sequence conservation (mathematically) and provides biological justifications. My aim here was to introduce the sampler to a biology-oriented audience, for whom such details cannot be described in a few sentences. Nevertheless, the short answer to your specific question is: The sampler does not down grade a conserved foreground position due to the specific nature of (divergent) background residues at that position. However, it can, at the same time, model the fact that the background subgroup conserves certain residues at the same position simply by including that subgroup in the hyperpartition. Note too that the sampler by no means requires fully conserved foreground pattern residues.

Reviewer 1

The fact that the most common consensus background residue in Figure 3A is leucine which is also one of the most common residues in databases would indicate that this background set approaches database composition for the most general comparisons while this is not the case for all other examples.

***Author's response**: This occurs because the background for this category is (mainly) represented by random sequences whose compositions are based on the overall distribution of amino acids within proteins*.

Reviewer 1

The lack of displaying representatives from the other subfamilies in the output alignment makes it difficult to verify the predicted status of subfamily-specific conservation. Since the seed alignment is curated and consists of only few members, it should be suitable to be displayed in the output as well as the main manuscript as figures including the annotation for positions with explicitly discussed subfamily-specific conservation. For example, the program Jalview allows conservation coloring based on group selections to highlight subfamily differences.

***Author's response**: I am not sure what the referee is requesting. Several subfamily alignments are given in Additional File *1*and the seed sequences are those explicitly shown in the alignments. Interested readers may download the GTPase input files and the mcBPPS sampler from my website to generate and examine all of the output alignments.*

Reviewer 1

While the structural part of the manuscript focuses on intra-molecular interpretation of the importance of the identified residues, the complexity of regulation through protein-protein interactions of various GTPases by other proteins is only marginally mentioned although it should play a major role in determining subfamily-specificities.

**Author's response**: I too more or less expected this. However, instead of driving an analysis via preconceived notions about what ought to be found, it seems best to allow the data itself to reveal its most striking properties. In this case, this appears to have led us in certain unexpected directions. One explanation for the scarcity of pattern residue interactions of the sort that you might have expected is that residues involved in specific protein-protein interactions have substantially diverged within these GTPase subfamilies; this is not so surprising considering that these subfamilies diverged from a common ancestor one to two billion years ago.

Reviewer 1

The argumentation of functional roles of residues based on existence of hydrogen bonds to known important residues is qualitatively interesting but several more residues in the vicinity that were not highlighted in the manuscript may fulfill the same criteria which raises the question if such arguments are truly specific. At the same time, the author goes into great detail to highlight the agreement with previous biochemical and structural studies which make this part of the description of the used dataset valuable as benchmark for comparison with other methods.

***Author's response**: I would be very surprised if other residues are not playing important roles. Many residues in the seed alignments are conserved across distinct phyla and thus presumably perform important functions. The purpose of a mcBPPS analysis thus is not to prove that these residues are the most important, but rather to obtain statistical clues regarding their biochemical roles. There are, of course, additional comparisons that could have been performed to help characterize other residues; this analysis is not intended to be exhaustive*.

Reviewer 1

Identification of subfamily-specific residues has been attempted multiple times by several different groups before and the manuscript would greatly benefit from mentioning different existing approaches. The list of references appended at the bottom of this referee report shows the broad spectrum of available methods and could be useful to any interested reader. To check robustness of the presented results over different methods or otherwise to highlight possible advantages of his approach, it would be good to apply 1 or 2 of the more successful alternative methods to the same dataset. It may be of interest to compare to results from methods that do not require a priori classified examples but rather calculate initial groupings from a phylogenetic tree to showcase advantages and disadvantages of different approaches on the GTPase dataset. Also, the question whether the enormous amount of aligned sequences is necessary to arrive at similar conclusions with methods that do not depend on large sample sizes could be addressed. Many of the observations may already be visible even in the seed alignment.

***Author's response**: I have added the references *[43-54], *as suggested. In addition, the forthcoming mcBPPS methods paper cites 16 such references, comments on the distinctions between the sampler and other methods, and evaluates the sampler's performance. Keeping the focus here on the sampler's biological application avoids both overlap with this companion paper and a lengthy discussion of the important distinctions between the sampler and other methods. For example, many of these methods seek to identify conserved residues directly responsible for substrate specificity. The sampler addresses a different computational problem, namely to identify (concurrently) both the most strikingly co-conserved residue patterns and the corresponding protein subgroups conserving those patterns--regardless of the biological functions of those residues. The goal is to obtain statistical clues regarding uncharacterized biochemical phenomena as an aid to biological discovery rather than to predict new instances of well understood phenomena. Statistical analyses are often used in this way to obtain preliminary information regarding biological processes that thus far have evaded detection due to inherent experimental limitations. For example, the hypothesis that components of tobacco smoke cause lung cancer in humans is (for obvious ethical reasons) still merely indirectly supported by statistical analyses.*

Reviewer 1

Overall, despite the above mentioned shortcomings that could be addressed in a revision, this manuscript provides a detailed view of functionally divergent residues in Arf/Arl GTPases and may become a good starting point for further studies, both in terms of the biology of GTPases as well as for improvement of computational methods to identify such residues.

***Author's response**: none required*.

### Reviewer 2: L Aravind, National Center for Biotechnology Information, National Institutes of Health, Bethesda, Maryland, USA

Reviewer 2

While a number of semi-manual approaches have been used by different researchers in identifying functionally relevant residues in alignments of large superfamilies, the method developed by Neuwald stands out in terms of the ability to use very large datasets (i.e. sequence alignments). This paper provides a powerful illustration of the automated Bayesian classification approach being applied to the Arf/Arl GTPases. The paper characterizes the mechanistic determinants of the interswitch toggle (i.e. the N-[VI] motif that is conserved within Arf/Arl GTPases). Further it goes on to attempt an explanation for the deviation from this pattern observed in the Arl8 GTPases. In the opinion of this referee the article is a very useful addition to our current understanding of GTPases and will certainly help future experimental studies on this important class of proteins.

***Author's response**: none required*.

#### Specific comments

Reviewer 2

-(p4) "An important subgroup of P-loop GTPases is the Ras-like superfamily [4] (termed here the Ras-like GTPases), members of which function as on-off switches within eukaryotic signaling pathways."

Given that the Ras-like superfamily exist in bacteria and archaea it would be advisable to make this statement more general to include them. The precursors of the Arf-like clade discussed here had already emerged in the bacteria.

***Author's response**: I have revised the paper accordingly*.

Reviewer 2

-(p8)" Indeed, less than 0.1% (224 out of 258,895) of the remaining P-loop GTPases identified in this analysis harbor an aspartate at this position--which is so low that it could be due merely to non-functional open reading frames and/or sequencing errors."

This statement that minority which harbor aspartate at this position may be non-function ORFs or sequencing errors does not seem to be entirely warranted. At least a few known to this referee seem to be good ORFs. It might be better to quickly check if these might belong to a particular distinct clade.

***Author's response**: I checked this, and you may be right; I have modified the text accordingly*.

Reviewer 2

-(p9-10) Is the P-loop glutamate truly equivalent to the aspartate of the Arf-like GTPases? I entirely accept the Gα-Arf-clade connection, and the fact that the Gα-Arf clade is defined among other things by the shared arginine in helix-3. However, the P-loop E in the Gαs appears to have emerged independently of the D in the Arfs. Thus, it is convergent similarity between the two. I suspect that this distinction between the two which might actually result in subtle mechanistic differences between them. This probably relates to the fact that the G#s have an in-built arginine finger whose function would be affected by the Arf-like condition.

***Author's response**: My main point was that both groups harbor a rather similar salt bridge between the P-loop and the α3 helix. I agree that the differences between the Gα and Arf/Arl salt bridges might reflect significant mechanistic differences. I have toned down the text accordingly*.

Reviewer 2

-(p13) "Arl8 GTPases conserve a glutamine instead of the P-loop aspartate typical of Arl/Arf/Sar GTPases and fail to conserve the arginine residue with which the P-loop aspartate interacts (not shown)."

Does this relate in any way to the differences in the Arl8 GAPs? What are the interaction partners of this Q residue?

***Author's response**: These are good questions, but there is not yet a structure of Arl8 bound to a GAP that could provide definitive answers*.

### Reviewer 3: Daniel Gaston Department of Biochemistry & Molecular Biology, Dalhousie University, Nova Scotia, Halifax, Canada (nominated by Eric Bapteste )

Reviewer 3

The submitted manuscript titled "Bayesian classification of residues associated with protein functional divergence: Arf and Arf-like GTPases" describes a novel Bayesian classifier for both the semi-supervised partitioning of a large multiple sequence alignment into a number of prespecified sub-groups (families with a protein superfamily, etc) as well as the classification of functionally divergent sites that maximally differentiate a group (foreground) from the rest of the sequences (background) in contrast alignments. This multiple category Bayesian Partitioning with Pattern Selection (mcBPPS) method is an extension of earlier published work of a BPPS classifier to a case of multiple categories instead of just two (foreground and background). The work demonstrates the utility of this method by analyzing a large multiple alignment (over 65,000 sequences) of P-loop GTPases, focusing particular attention on the Raslike superfamily and in particular on the Arf/Arl/SAR family and G-alpha subunits. A number of differentially conserved residues are identified that, based on further structural evidence and analysis based on the author's CHAIN method, seem like good candidates for explaining the functional differences between different functional sub-types.

***Author's response**: none required*.

#### General comments

Reviewer 3

This work should be broad appeal to molecular biologists and importantly is able to handle a very large number of sequences. In earlier work the author points out that this is important to get away from potential sampling biases when performing analyses of functional divergence. While this is true I would suggest that breadth of phylogenetic sampling has perhaps the highest impact on these types of analyses and that a large number of sequences in this dataset are likely highly redundant, coming from very closely related taxa. While the seed alignments are claimed to be phylogenetically diverse, constituting representative sequences from different phyla, the large number of sequences that make up the final optimal alignments for each group from NCBI sources may give false impressions of the level of sequence conservation at a given site due to sampling bias in the databases themselves. Certain very highly studied groups and organisms are over represented in sequence databases.

***Author's response**: The issue of sequence redundancy was dealt with in two ways: First, nearly identical sequences (as well as sequence fragments) were removed; this pruned the input alignment from ~259,000 down to ~65,000 sequences. Second, sequences in the alignment are down weighted for redundancy using the weighting scheme used by PSI-BLAST. These and other mcBPPS methodological issues are discussed in previous publications and in the statistically- and algorithmically-oriented methods paper to be published separately. These measures also address to some degree the issue of sampling bias--which is also addressed by performing multiple comparisons from various perspectives and using various input alignments either that were constructed independently or from which significant numbers of sequences were randomly removed. The forthcoming methods paper includes such evaluations*.

**Reviewer's post-revision remark: **Author's response addresses my concerns regarding comparisons to other programs. Would personally prefer some information in supplementary material only because the methods paper is not yet accepted/in press, but focus on the biological findings is appropriate.

Reviewer 3

Visualizing the phylogenetic context of these subgroups, families, and superfamilies would help clarify the discussion of conserved/non-conserved residue positions in the main body of the work. While the hyperpartition in Figure 2 gives some indication, showing the Arf/Arl/SAR group located within the Ras-like superfamily for instance, no real indication of the relationship of groups within this family is given (Such as the relationship between Arl6 and Arl8 with the rest of the Arf/Arl/SAR family for instance). I would highly suggest a schematic tree representation that corresponds to the hyperpartition shown in Figure 2 showing these phylogenetic relationships. Some indication of the number of sequences in the seed alignment for each group, as well as the total number of sequences after the optimal partitioning scheme is determined, would also be helpful and clarifying. What phyla are represented in the seed alignments (perhaps as supplementary material) would also be helpful.

***Author's response**: As is clear from reference *[4], *there is considerable uncertainty about the precise phylogenetic relationships between various P-loop GTPases. Hence, the validity of such a phylogenetic tree is questionable. I have added to Additional File *1*the requested information regarding the numbers of seed sequences and the phyla they represent.*

Reviewer 3

While the basis for this method has already been published, in general when new methods are proposed some level of comparison to other existing methods would be ideal. While this Bayesian classifier handles a much larger number of sequences than most functional divergence prediction programs can (this dataset in particular), why should molecular biologists use this particular method as opposed to others that have been published such as DIVERGE [55], GroupSim [48], Multi-RELIEF [56], or similar? The answer is obvious for some of these in that the new method does semi-automated partitioning of sequences and can handle a very large number of sequences. But does this method predict any critical residues in this dataset that are missed by other programs? Does it produce fewer likely false-positives? Some sort of comparison would be helpful in convincing the reader that this proposed method would be the most useful for them and that it provides high quality predictions.

***Author's response**: See my response to Reviewer 1's similar comment above*.

Reviewer 3

Overall I think that this analysis is very thorough and that the introduced method will be useful to a broad segment of the molecular biology community.

***Author's response**: none required*.

#### Specific comments

Reviewer 3

I would recommend that the discussion of the Bayesian method (Introduction starting in paragraph 4) be restructured somewhat to indicate from the initial discussion that seed alignments will be used (paragraph 7). For a general molecular biology reader this description could be streamlined a bit more as further information is provided in the appropriate references.

***Author's response**: I have added a comment regarding the use of seed sequences, as requested. Referee 1's suggestion to add a few more methodological details appears to conflict with this referee's streamlining suggestion. Nevertheless, I have streamlined this section somewhat for clarity*.

Reviewer 3

In discussions of residues differentially conserved within, for instance, the Arf/Arl/SAR group versus the rest of the Ras superfamily it would make discussion clearer to remind readers of this nested superfamily/family structure. "Residue X is conserved within the Arf/Arl/SAR GTPases while in the rest of the Ras-like GTPases it is Y..." for instance.

**Author's response**: In most cases, a variety of residues are conserved in the background sequences; however, I have made an effort to include such statements when pertinent.

Reviewer 3

Features Distinguishing Arf/Arl/Sar from other P-loop GTPases paragraph 5 (Similarities with G-alpha subunits). This section would be clarified in terms of interpretation somewhat with the addition of the proper phylogenetic context as discussed above since the G-alpha subunits conserve this glycine residue that is putatively the ancestral state for all Ras-like GTPases. Mentions a conserved P-loop glutamate immediately following this glycine, what is this residue in the other Arf/Arl/Sar sequences? Is it just non-conserved?

**Author's response**: As mentioned above, the precise phylogeny of these GTPases is uncertain, as is the ancestral status of the P-loop glycine. Although the Ras, Rho, Ran and Gα families conserve a glycine at this position, the Rab family, which best conserves the Ras-like canonical features, most often conserves a serine at this position. The residue corresponding to the Gα P-loop glutamate is typically an asparagine in Arf/Arl/Sar GTPases. Taking the analysis deeper in this and numerous other ways would no doubt be informative, but seems best left for future studies. Researchers working on these proteins may download the sampler and the data in order to explore those questions most relevant to their research.

Reviewer 3

Paragraph 6 of same section. Salt Network versus Glycine Brace discussion. Mention a distinguishing feature (Phe90) that only shows up under low contrast settings. What are these contrast settings used in the regular analysis and what is the reduced setting used to have this feature show up? How many other putative distinguishing features/functionally divergent sites show up under these more liberal conditions? Some brief discussion of the contrast settings would be appropriate in the description of the method earlier in the manuscript, perhaps with expanded detail in supplementary material. Similarly a brief description of the quantified "evolutionary constraint" that appears as histogram above sequences in Figure 3 with ref to Neuwald et al, 2003 for further details.

***Author's response**: Detailed information regarding the contrast setting is found in Appendix 1 of the CHAIN program user's manual, which is available at http://www.chain.umaryland.edu. Briefly, the contrast setting determines how many of the most discriminating pattern residues are highlighted in the output alignment. The histograms in Figure *3*merely provide a qualitative measure of the relative constraints, and thus cannot be described quantitatively. The mcBPPS sampler outputs a pattern information file which provides quantitative measures corresponding to these histograms. This measure corresponds to the contribution (in nats) of individual pattern positions to the underlying statistical model's posterior log-likelihood ratio*.

## Supplementary Material

Additional file 1 **Output alignments, numbers of sequences assigned to each subgroup, numbers and phyla of seed sequences**. Includes Figures S1 to S5. Click here for file

Additional file 2 **The Arf/Arl/Sar salt bridge network and the retinitis pigmentosa 2 (RP2) protein, a GAP for Arl3**. Analysis of RP2-related proteins using the mcBPPS sampler. Click here for file
