# Supplementary material for: Bayesian classification of residues associated with protein functional divergence: Arf and Arf-like GTPases
Source: Biol Direct. 2010 Dec 3;5:66. doi: 10.1186/1745-6150-5-66 (PMC3012027; doi:10.1186/1745-6150-5-66)

Additional file 1 for

Bayesian classification of residues associated with protein functional divergence: Arf and Arf-like GTPases

Andrew F. Neuwald

Output alignments.

Figure legends

Figure S1. The mc-BPPS output for Rab GTPases (row 10 of Fig. 2). **(A)** Contrast alignment highlighting distinguishing features of the TRAFAC subclass of P-loop GTPases. **(B)** Contrast alignment highlighting Ras-like distinguishing features. **(C)** Contrast alignment highlighting distinguishing features of Rab/Ran/Rho GTPases.

Figure S2. Output alignments for Arf1 GTPases (row 2 in Fig. 2). **(A)** Contrast alignment highlighting distinguishing features of the TRAFAC subclass of P-loop GTPases. **(B)** Contrast alignment highlighting Ras-like distinguishing features. **(C)** Contrast alignment highlighting Arf/Arl/Sar distinguishing features. **(D)** Contrast alignment highlighting Arf/Arl distinguishing features. **(E)** Contrast alignment highlighting Arf1 distinguishing features.

Figure S3. Output alignments for Sar GTPases (row 9 in Fig. 2). **(A)** Contrast alignment highlighting distinguishing features of the TRAFAC subclass of P-loop GTPases. **(B)** Contrast alignment highlighting Ras-like distinguishing features. **(C)** Contrast alignment highlighting Arf/Arl/Sar distinguishing features. **(D)** Contrast alignment highlighting Sar distinguishing features.

Figure S4. Output alignments for Arl8 GTPases (row 6 in Fig. 2). **(A)** Contrast alignment highlighting distinguishing features of the TRAFAC subclass of P-loop GTPases. **(B)** Contrast alignment highlighting Ras-like distinguishing features. **(C)** Contrast alignment highlighting Arf/Arl/Sar distinguishing features. **(D)** Contrast alignment highlighting Arl8 distinguishing features.

Figure S5. Output alignments for RP2 related proteins (row 1 of Table S2). **(A)** Contrast alignment highlighting distinguishing features of all RP2-related proteins. **(B)** Contrast alignment highlighting distinguishing features of RP2 versus Tubulin-folding cofactor C proteins. **(C)** Contrast alignment highlighting distinguishing features of RP2 and Tubulin-folding cofactor C GAP domains versus other, more distantly-related domains.

Numbers of sequences assigned to each subgroup.

===================== HyperPartition: =====================

Category

Set:	1	2	3	4	5	6	7	8	9	10	11	12	13	14	15	16	17	18	19	20	21	
1:	+	-	+	-	-	+	-	-	-	-	-	o	-	-	-	-	-	-	-	-	+	Galpha (384)
2:	+	-	+	-	-	+	-	-	-	-	-	+	+	-	-	-	-	-	-	+	-	Arf1 (1093)
3:	+	-	+	-	-	+	-	-	-	-	-	+	+	-	-	-	-	-	+	-	-	Arl2 (254)
4:	+	-	+	-	-	+	-	-	-	-	-	+	+	-	-	-	-	+	-	-	-	Arl5 (186)
5:	+	-	+	-	-	+	-	-	-	-	-	+	o	-	-	o	+	-	-	-	-	Arl6 (71)
6:	+	-	+	-	-	+	-	-	-	-	-	+	o	-	-	+	o	-	-	-	-	Arl8 (364)
7:	+	-	+	-	-	+	-	-	-	-	-	+	+	-	+	-	-	-	-	-	-	Arl1 (166)
8:	+	-	+	-	-	+	-	-	-	-	-	+	+	-	-	-	-	-	-	-	-	MiscArl (742)
9:	+	-	+	-	-	+	-	-	-	-	-	+	-	+	-	-	-	-	-	-	-	Sar (570)
10:	+	-	+	-	-	+	-	-	-	+	+	-	o	o	o	o	o	o	o	o	o	Rab (5858)
11:	+	-	+	-	-	+	-	-	+	-	+	-	o	o	o	o	o	o	o	o	o	Ran (449)
12:	+	-	+	-	-	+	-	+	-	-	+	-	o	o	o	o	o	o	o	o	o	RhoLike (1507)
13:	+	-	+	-	-	+	+	-	-	-	o	-	o	o	o	o	o	o	o	o	o	Ras (824)
14:	+	-	+	-	-	+	-	-	-	-	o	-	o	o	o	o	o	o	o	o	o	MiscRasLike (1793)
15:	+	-	+	-	+	-	o	o	o	o	o	-	o	o	o	o	o	o	o	o	o	EFlike (12199)
16:	+	-	+	+	-	-	o	o	o	o	o	-	o	o	o	o	o	o	o	o	o	ObgSF (15293)
17:	+	-	+	-	-	-	o	o	o	o	o	-	o	o	o	o	o	o	o	o	o	MiscTRAFAC (9656)
18:	+	+	o	-	-	o	o	o	o	o	o	-	o	o	o	o	o	o	o	o	o	SIMIBI (10821)
19:	-	-	-	-	-	o	o	o	o	o	o	o	o	o	o	o	o	o	o	o	o	Rejected (4156)

Numbers and phyla of seed sequences.

Rab(11 seqs): num_phyla = 3
Apicomplexa (1)
===== 1 protozoa (E) =====
Ascomycota (3)
===== 3 fungi (F) =====
Chordata (7)
===== 7 metazoa (M) =====

Ran(18 seqs): num_phyla = 18
Alveolata (1)
Apicomplexa (1)
Cryptophyta (1)
Entamoebidae (1)
Euglenozoa (1)
Mycetozoa (1)
Rhodophyta (1)
stramenopiles (1)
===== 8 protozoa (E) =====
Ascomycota (1)
Chytridiomycota (1)
===== 2 fungi (F) =====
Arthropoda (1)
Chordata (1)
Cnidaria (1)
Ctenophora (1)
Echinodermata (1)
Mollusca (1)
Nematoda (1)
Platyhelminthes (1)
===== 8 metazoa (M) =====

Sar(15 seqs): num_phyla = 14
Alveolata (1)
Apicomplexa (1)
Entamoebidae (1)
Euglenozoa (1)
Mycetozoa (1)
stramenopiles (1)
===== 6 protozoa (E) =====
Ascomycota (1)
Basidiomycota (1)
===== 2 fungi (F) =====
Arthropoda (1)
Chordata (2)
Echinodermata (1)
Nematoda (1)
Platyhelminthes (1)
===== 6 metazoa (M) =====
Streptophyta (1)
===== 1 plants (V) =====

RhoLike(5 seqs): num_phyla = 3
Arthropoda (1)
Chordata (3)
Mollusca (1)
===== 5 metazoa (M) =====

MiscRasLike(1 seqs)
Consensus seq (1)

Arf1(16 seqs): num_phyla = 16
Alveolata (1)
Apicomplexa (1)
Bacillariophyta (1)
Cercozoa (1)
Diplomonadida group (1)
Entamoebidae (1)
Euglenozoa (1)
Haptophyceae (1)
Mycetozoa (1)
===== 9 protozoa (E) =====
Basidiomycota (1)
===== 1 fungi (F) =====
Annelida (1)
Chordata (1)
Echinodermata (1)
Tardigrada (1)
===== 4 metazoa (M) =====
Chlorophyta (1)
Streptophyta (1)
===== 2 plants (V) =====

Arl2(10 seqs): num_phyla = 10
Alveolata (1)
Mycetozoa (1)
===== 2 protozoa (E) =====
Basidiomycota (1)
===== 1 fungi (F) =====
Arthropoda (1)
Chordata (1)
Cnidaria (1)
Echinodermata (1)
Nematoda (1)
Platyhelminthes (1)
===== 6 metazoa (M) =====
Streptophyta (1)
===== 1 plants (V) =====

Arf6(5 seqs): num_phyla = 3
Arthropoda (1)
Chordata (3)
Nematoda (1)
===== 5 metazoa (M) =====

Arl5(10 seqs): num_phyla = 9
Fungi/Metazoa group (1)
Mycetozoa (1)
===== 2 protozoa (E) =====
Arthropoda (1)
Chordata (2)
Cnidaria (1)
Echinodermata (1)
Nematoda (1)
Platyhelminthes (1)
===== 7 metazoa (M) =====
Streptophyta (1)
===== 1 plants (V) =====

Arf5(4 seqs): num_phyla = 1
Chordata (4)
===== 4 metazoa (M) =====

Arl1(13 seqs): num_phyla = 13
Apicomplexa (1)
Euglenozoa (1)
stramenopiles (1)
===== 3 protozoa (E) =====
Ascomycota (1)
Basidiomycota (1)
Chytridiomycota (1)
===== 3 fungi (F) =====
Arthropoda (1)
Chordata (1)
Echinodermata (1)
Nematoda (1)
Tardigrada (1)
===== 5 metazoa (M) =====
Chlorophyta (1)
Streptophyta (1)
===== 2 plants (V) =====

MiscArl(6 seqs): num_phyla = 2
Ascomycota (1)
===== 1 fungi (F) =====
Chordata (5)
===== 5 metazoa (M) =====

Galpha(10 seqs): num_phyla = 1
Chordata (10)
===== 10 metazoa (M) =====

EFlike(26 seqs): num_phyla = 0
Consensus seqs (26)

ObgSF(22 seqs): num_phyla = 0
Consensus seqs (22)

SIMIBI(12 seqs): num_phyla = 0
Consensus seqs (12)

MiscTRAFAC(1 seq): phyla = 0
Consensus seq (1)

Ras(11 seqs): num_phyla = 11
Apicomplexa (1)
Mycetozoa (1)
===== 2 protozoa (E) =====
Ascomycota (1)
Basidiomycota (1)
Zygomycota (1)
===== 3 fungi (F) =====
Arthropoda (1)
Chordata (1)
Cnidaria (1)
Nematoda (1)
Platyhelminthes (1)
===== 5 metazoa (M) =====
Embryophyta (1)
===== 1 plants (V) =====

Arl8(8 seqs): num_phyla = 8
Annelida (1)
Arthropoda (1)
Chordata (1)
Cnidaria (1)
Echinodermata (1)
Nematoda (1)
Platyhelminthes (1)
===== 7 metazoa (M) =====
Streptophyta (1)
===== 1 plants (V) =====

Arl6(8 seqs): num_phyla = 8
stramenopiles (1)
===== 1 protozoa (E) =====
Arthropoda (1)
Chordata (1)
Cnidaria (1)
Mollusca (1)
Platyhelminthes (1)
===== 5 metazoa (M) =====
Chlorophyta (1)
Streptophyta (1)
===== 2 plants (V) =====

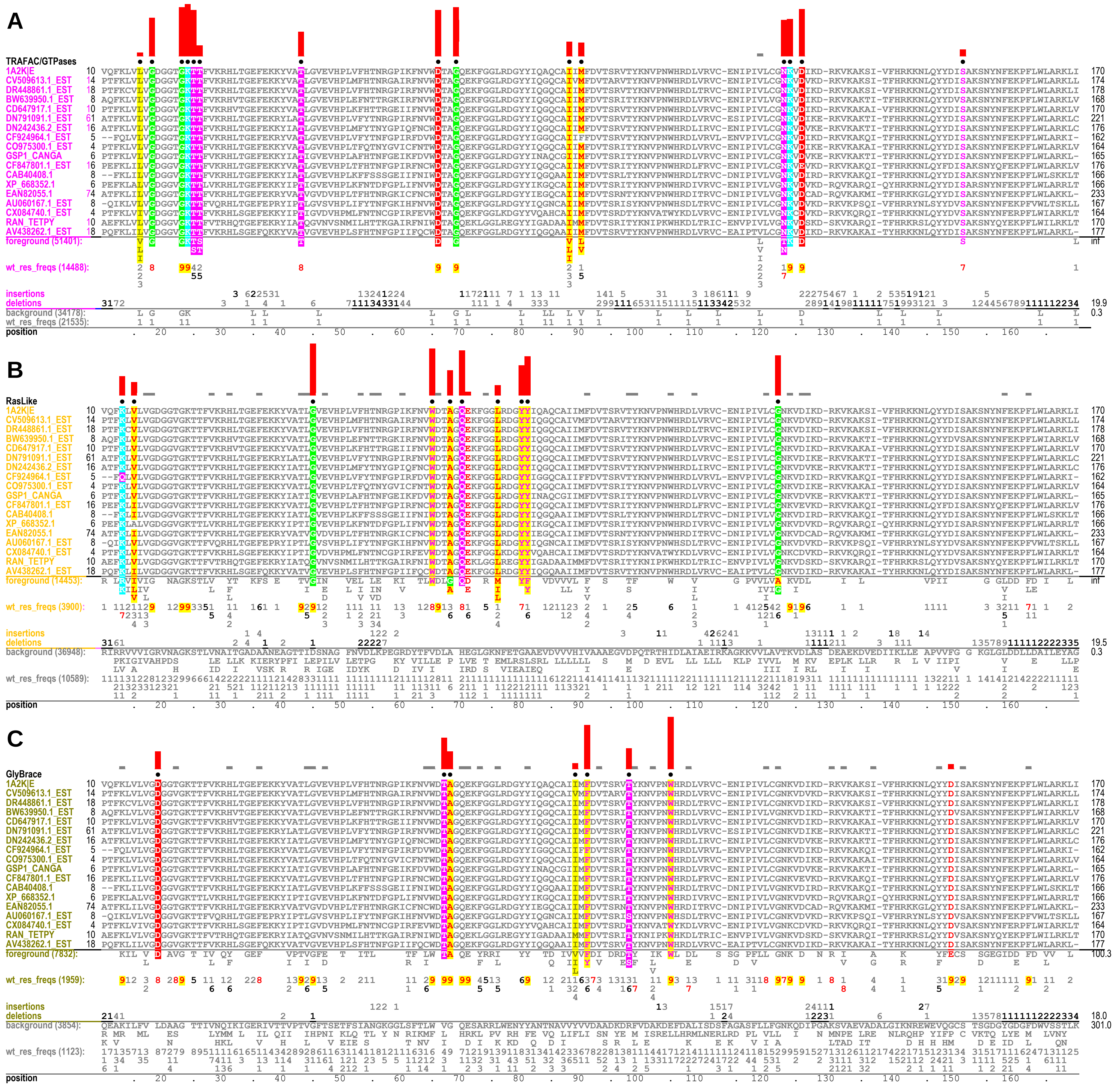
A
TRAFAC/GTPases
B
RasLike
C
GlyBrace

Neuwald Fig. S2

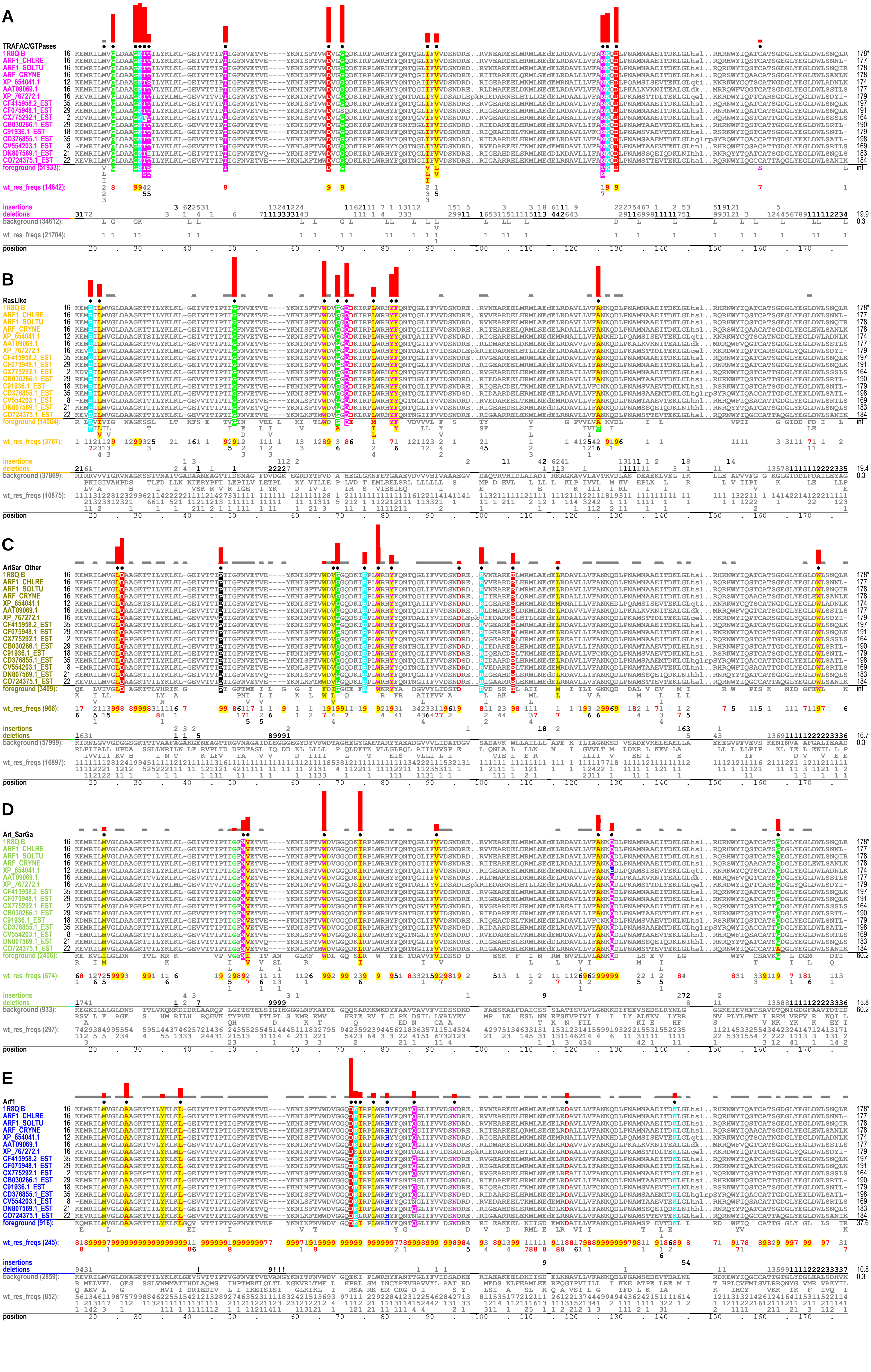

Neuwald, Fig. S3

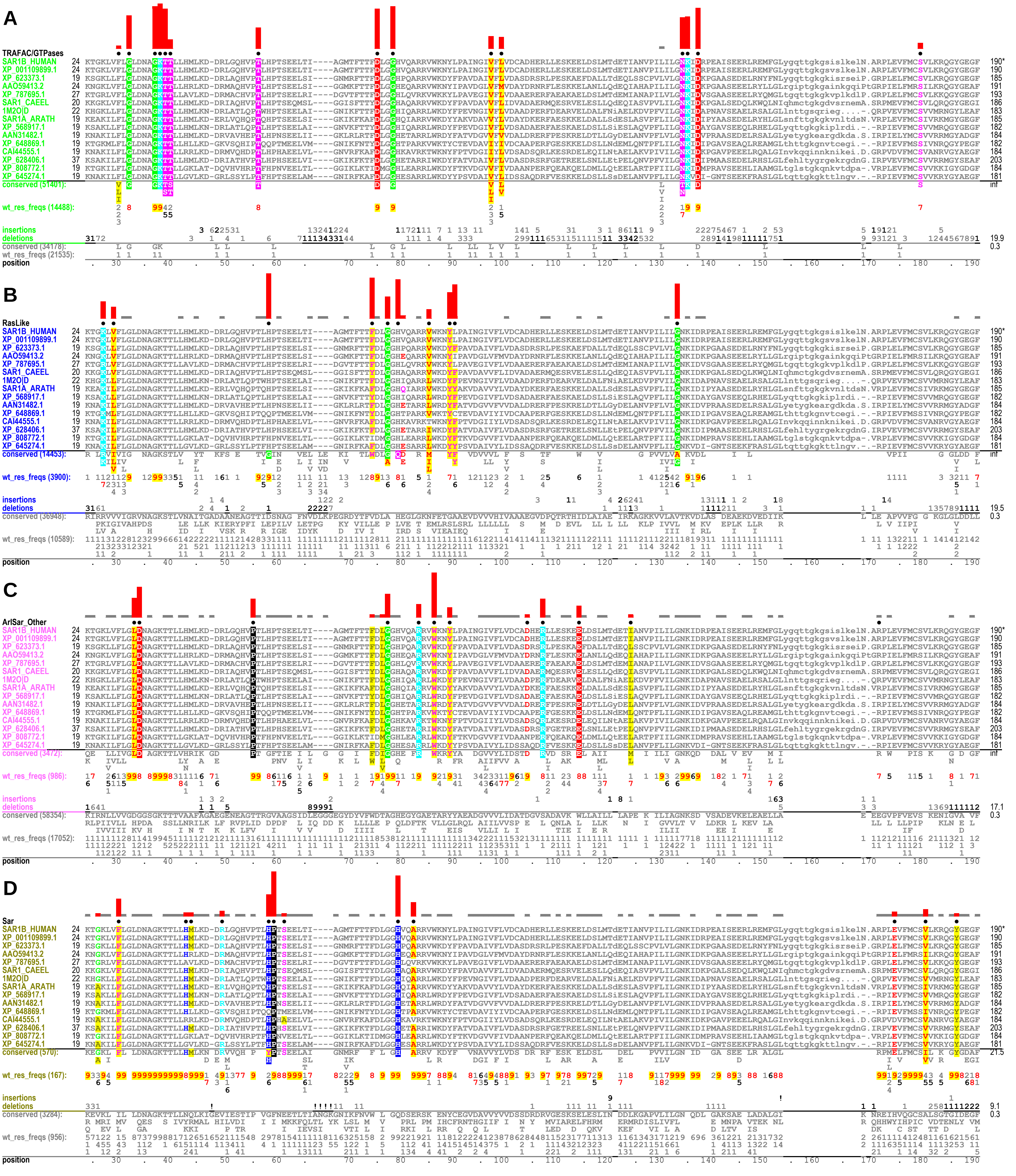

Neuwald, Fig. S4

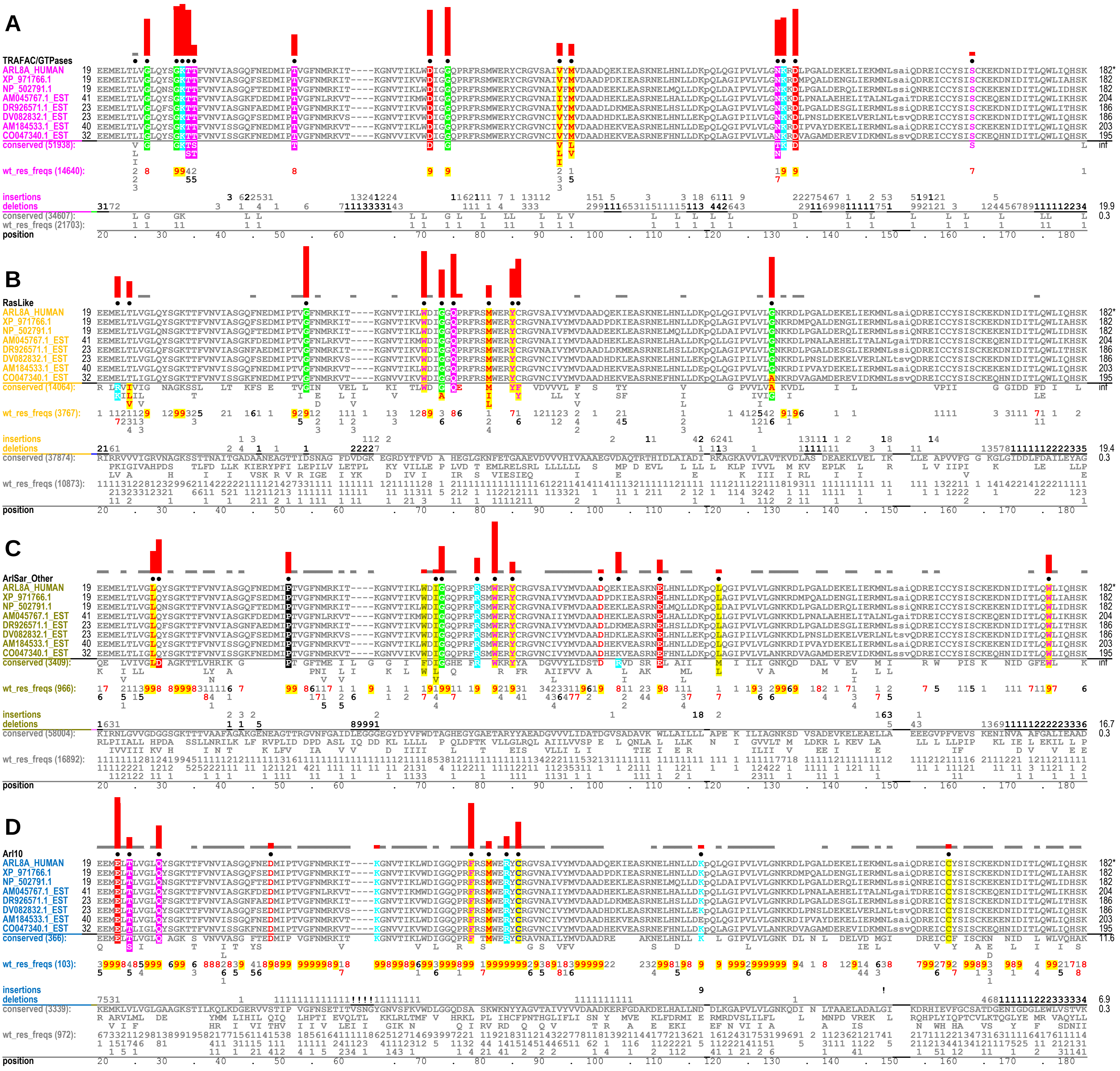

Neuwald Figure S5

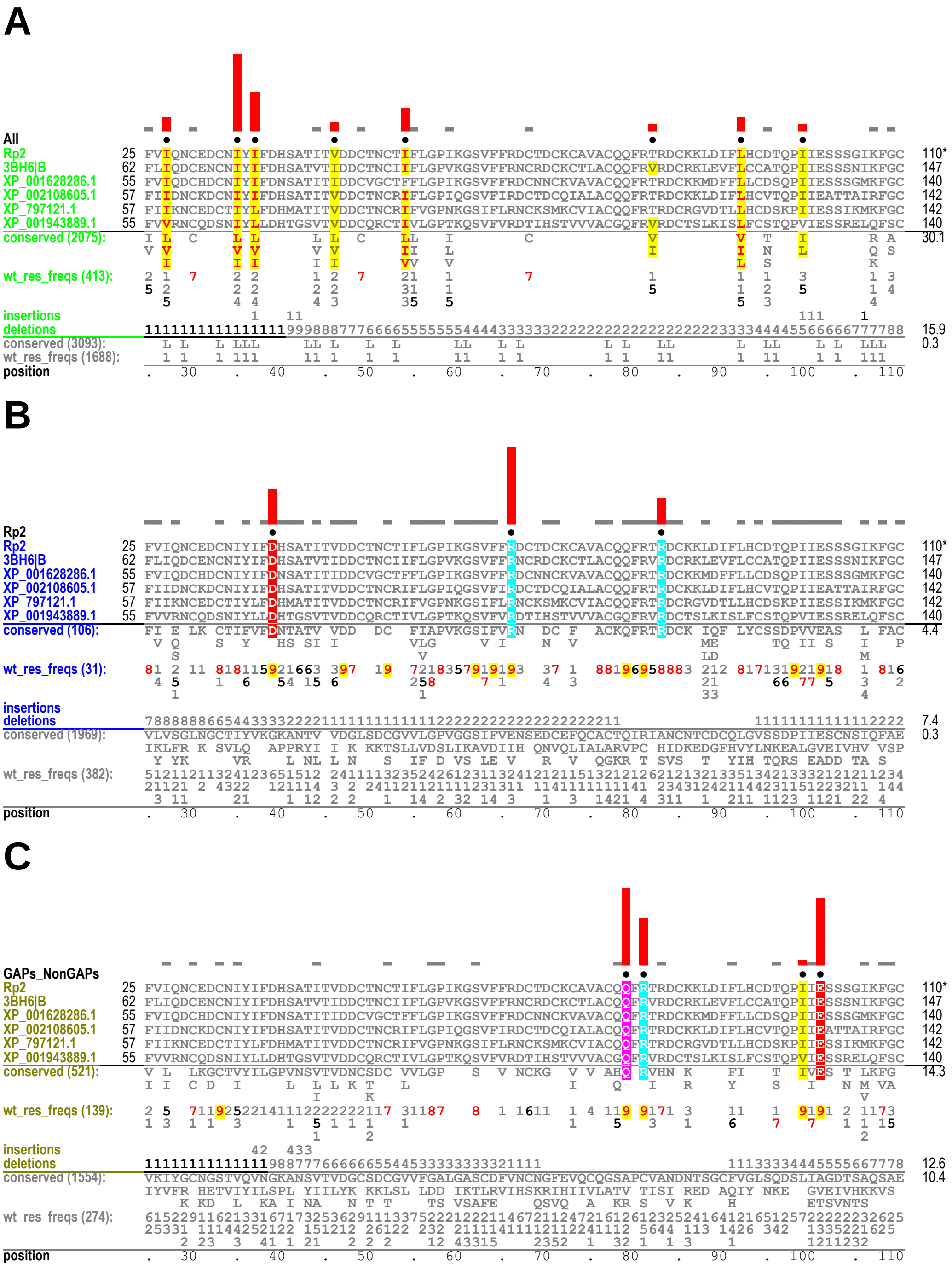

Supplement: Additional file 1 — Output alignments, numbers of sequences assigned to each subgroup, numbers and phyla of seed sequences. Includes Figures S1 to S5. [file 1745-6150-5-66-S1.PDF]
