# Supplementary material for: Bayesian classification of residues associated with protein functional divergence: Arf and Arf-like GTPases
Source: Biol Direct. 2010 Dec 3;5:66. doi: 10.1186/1745-6150-5-66 (PMC3012027; doi:10.1186/1745-6150-5-66)
Supplement: Additional file 2 — The Arf/Arl/Sar salt bridge network and the retinitis pigmentosa 2 (RP2) protein, a GAP for Arl3. Analysis of RP2-related proteins using the mcBPPS sampler. [file 1745-6150-5-66-S2.PDF]

Additional file 2 for

Bayesian classification of co-conserved residues associated with protein functional divergence: Arf and Arf-like GTPases

Andrew F. Neuwald

The Arf/Arl/Sar salt bridge network and the retinitis pigmentosa 2 (RP2) protein, a GAP for Arl3.

A mc-BPPS analysis was performed on the product of the retinitis pigmentosa 2 gene (termed RP2), which functions as a GTPase-activating protein for Arl3 GTPase [20]. The hyperpartition is shown in Table S2 and corresponding contrast alignments for RP2 in Fig. S2-1. Within the transition state structure of Arl3 bound to RP2 the Arf/Arl/Sar co-conserved residues of Arl3 (yellow side chains in Fig. S2-2) form a network of hydrogen bonds that appear to help position the P-loop aspartate (Asp26-Arl3 in Fig. S2-2) and the Arf/Arl Walker B glycine (Gly70-Arl3) relative to the AlF_4 molecule, which mimics the transitional form of the leaving γ-phosphate group. Indeed, in this structure the P-loop aspartate backbone conformation is very similar to that of Gly12 within the Ras-RasGAP complex[59](not shown). At the same time, interactions involving residues that are specifically conserved within RP2 also appear to help precisely position certain Arf/Arl residues within the transitional state. Two categories of residues specifically conserved within RP2 were identified in the mc-BPPS analysis shown in Fig. S2-1. The first category of residues (Fig. S2-1A; cyan side chains in Fig. S2-2, top inset) are conserved within RP2, but not within Tubulin-folding cofactor C (also called Tubulin binding cofactor C or TBCC), a GAP that is closely related to and that functionally overlaps with RP2 [60]. The most strikingly characteristic residues within this category are: (i) an arginine (Arg103-RP2 in Fig. S2-2, top inset) that forms a hydrogen bond with the Arf/Arl aspartate residue within the α3-helix (Asp96-Arl3); and (ii) an aspartate (Asp84-RP2) that hydrogen bonds to this RP2 arginine.

A second category of RP2 residues (Fig. S2-1B; green side chains in Fig. S2-2) are those conserved both in RP2 and in TBCC, but not within TBCC-related domains that lack an arginine finger characteristic of GAPs and that thus presumably lack GAP activity. The latter includes, for example, adenylyl cyclase-associated proteins [61]. The most strikingly characteristic residues in this category are: (i) the arginine finger (Arg118-RP2), which packs up against the Arf/Arl-conserved proline (Pro47-Arl3) and which hydrogen bonds to a water molecule that, in turn, forms a hydrogen bond to the Arf/Arl P-loop aspartate (Asp26-Arl3); (ii) a glutamine (Gln116-RP2) that hydrogen bonds to the arginine finger; and (iii) a glutamate (Glu138-RP2) that forms a hydrogen bond to a water molecule that, in turn, forms a hydrogen bond to the Arf/Arl P-loop aspartate and that establishes an electrostatic interaction with the positive dipole moment associated with the switch II helix. Note too that the arginine finger of RP2 packs against the Arf/Arl/Sar-conserved proline residue (Pro47) just as the Sec23 arginine finger packs against the corresponding proline in Sar1 (Pro53 in Fig. 4F of the paper); this interaction may explain why this proline is conserved.

Thus, interactions between certain RP2 residues and Arf/Arl/Sar -specific residues within Arl3 appear to help precisely position key residues near the γ-phosphate so as to facilitate GTP hydrolysis. Note,

however, that GAPs for other Arf/Arl/Sar family members conserve different features that presumably reflect distinct mechanisms facilitating GTP hydrolysis. For example, in a (non-transition state) structure of Sar1 [41] bound to a GTP analog and to its GAP, Sec23 (Fig. 4F), the GAP arginine finger (Arg722-Sec23) forms a hydrogen bond with the side chain oxygen of a conserved serine (Ser719-Sec23) that, in turn, forms a hydrogen bond with the P-loop aspartate. Likewise, the structure of Arf6 bound to a GAP belonging to the ASAP subfamily [62] indicates yet another, calcium-regulated mechanism.

Sequences and structures used in this analysis.

Sequences were identified within the NCBI nr, env_nr and translated EST databases; only translated EST open reading frames of at least 100 residues in length were used. The database identifiers (and phyla) for the RP2 and RP2-like sequences in Fig. S2-1 are: RP2_mouse, pdb_id=3BH6B (Chordata); sea anemone, gi=156370052 (Cnidaria); *Trichoplax*, gi=195997473 (Placozoa); purple sea urchin, gi=72138972, (Echinodermata); pea aphid, gi=193697819, (Arthropoda).

Table S2. Hyperpartition for RP2-related proteins.

Categories[1]	Subgroup
+ − − + + +	Rp2
+ − + − − +	TBCC
+ + − − o −	CAP
+ − − − o −	*MiscTBCC*
− o o o o o	Rejected[2]

[1]The symbols '+', '-' or 'o' indicate that the subgroup is assigned to that column's foreground, background, or non-participating-sequence partition, respectively. [2]Rejected sequences are those assigned with random sequences.

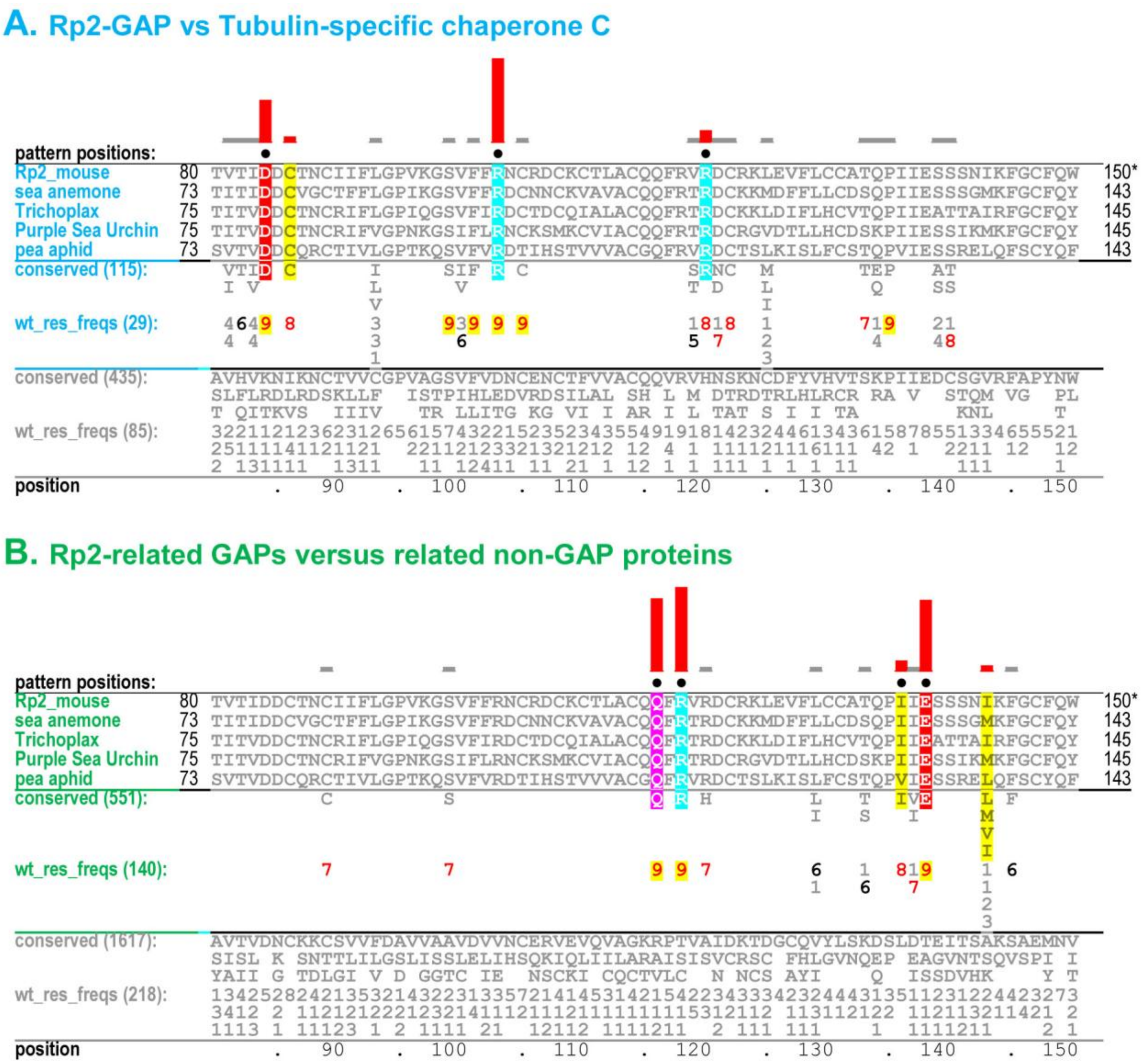

Figure S2-1. Contrast alignments of the GAP protein retinitis pigmentosa 2 (RP2). **(a)** Contrast alignment of RP2 versus Tubulin-folding cofactor C proteins (column 5 in Table S2). **(b)** Contrast alignment of RP2 and Tubulin-folding cofactor C putative GAP domains versus other, more distantly-related domains (column 6 in Table S2).

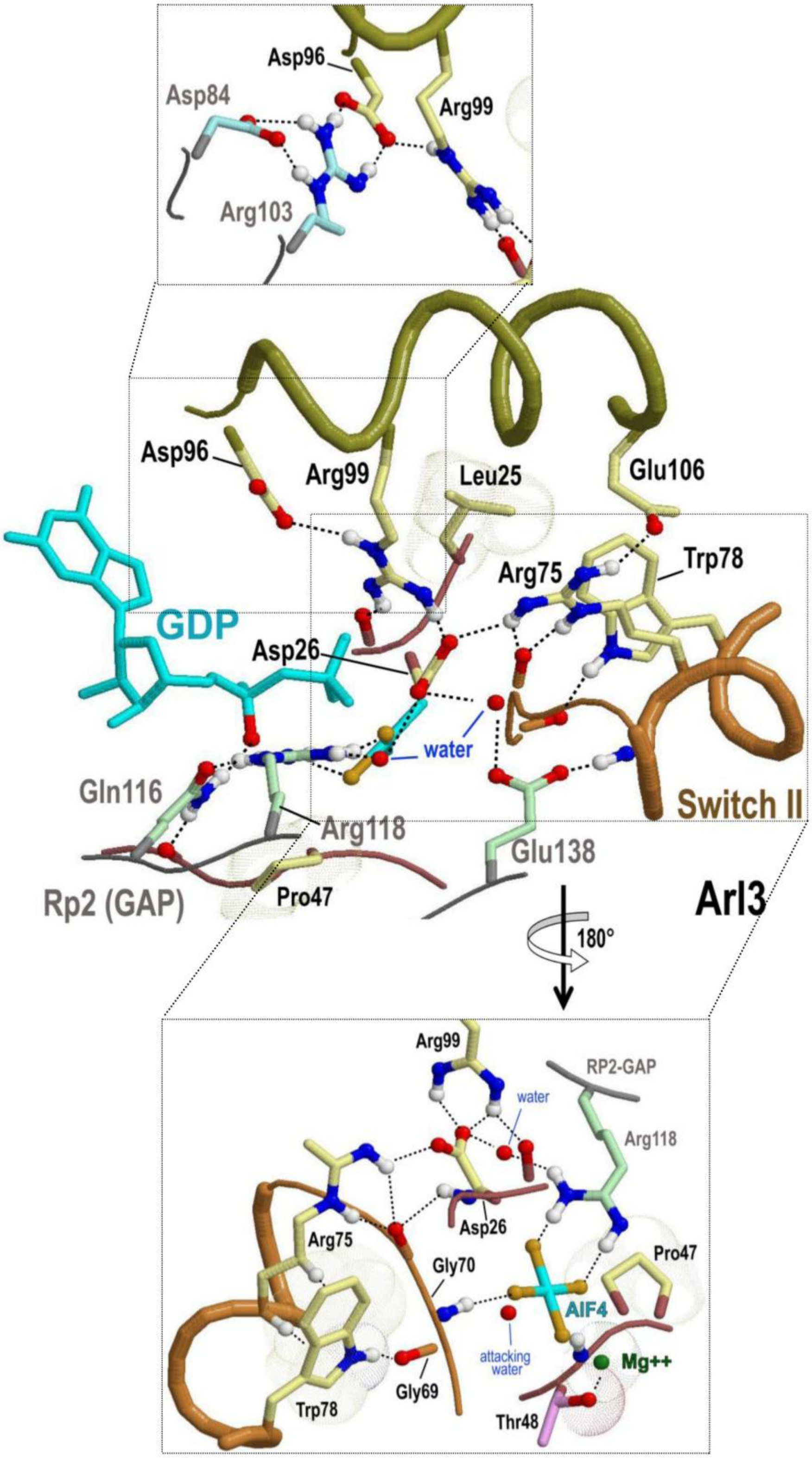

Figure S2-2. Distinguishing features of Arf/Arl/Sar GTPases and of retinitis pigmentosa 2 (RP2) within the structure of Arl3 bound to RP2 (a GAP for Arl3), to GDP and to the γ-phosphate transition state mimic AlF_4 (pdb: 3bh7; 1.90 Å) [20]. Shown are key interactions between Arf/Arl/Sar co-conserved residues (yellow side chains) within Arl3 and residues highly conserved within RP2. Shown in cyan (top inset) are side chains of residues that are highly conserved within RP2 but non-conserved in the related GAP domain of Tubulin-folding cofactor C (Fig. S2-1A). Shown in green are side chains of residues characteristic of RP2-related GAPs but not in other RP2-related domains (Fig. S2-1B). Top inset: Interaction of RP2 with the N-terminal end of the Arl3 α3 helix. Bottom inset: key interactions near the γ-phosphate pocket of the Arl3-RP2 complex.
